# Reduced Maintenance DNA Methylation Thresholds Enable Sensitive Reporter Assays for UHRF1 and DNMT1 Inhibition

**DOI:** 10.1002/advs.202519080

**Published:** 2026-03-26

**Authors:** Cuicui Xia, Ying Cui, Jiongliang Wang, Wenjing Bai, Danyang Wang, Yao Chen, Huahui Guo, Liangyi Zong, Bing Qin, Xintong Cai, Jie Wang, Jing Huang, Scott B. Rothbart, Zufeng Guo, Stephen B. Baylin, Xiangqian Kong

**Affiliations:** ^1^ Division of Life Sciences and Medicine University of Science and Technology of China Hefei China; ^2^ Institute of Drug Discovery Guangdong Provincial Key Laboratory of Stem Cell and Regenerative Medicine China‐New Zealand Joint Laboratory on Biomedicine and Health Guangzhou Institutes of Biomedicine and Health Chinese Academy of Sciences Guangzhou China; ^3^ The Sidney Kimmel Comprehensive Cancer Center at Johns Hopkins The Johns Hopkins University School of Medicine Baltimore Maryland USA; ^4^ College of Pharmacy Basic Medicine Research and Innovation Center For Novel Target and Therapeutic Intervention (Ministry of Education) Department of Breast and Thyroid Surgery of the Second Affiliated Hospital Chongqing Medical University Chongqing China; ^5^ Department of Epigenetics Van Andel Institute Grand Rapids Michigan USA

**Keywords:** DNA hypomethylating agents, DNMT1 inhibition, HTS assay development, recombinant reporter assay, UHRF1 inhibition

## Abstract

Aberrant DNA methylation and consequent silencing of tumor suppressor gene (TSGs) are maintained by UHRF1‐mediated recruitment of DNMT1, making the UHRF1‐DNMT1 axis as an attractive target for hypomethylating agents (HMAs). However, fully reversing DNA methylation abnormalities requires lowering DNMT1 below a deep threshold, posing major drug discovery challenges. Here, we demonstrated that a similarly stringent threshold applied to UHRF1 in maintaining hypermethylated TSG suppression. Genetically reducing redundancy in DNMT1 or UHRF1 markedly lowered these thresholds, sensitizing cells to their inhibition. These findings were translated into engineered recombinant reporter systems tied to endogenous hypermethylated promoters in hypomorphic cells, substantially improving sensitivity and dynamic range for monitoring HMA‐induced TSG reactivation. Pilot high‐throughput screening validated assay robustness and specificity for DNMT1 and UHRF1 inhibitors. Additionally, these platforms enabled optimization of timing and combination strategies pairing DNMT1/UHRF1 inhibitors with other epigenetic agents to maximize TSG re‐expression. Our study supports the concept of maintenance methylation thresholds and provides versatile tools for discovering novel HMAs and designing epigenetic combination therapies.

## Graphical abstract

The authors engineer colorectal cancer cells with reduced redundancy of UHRF1 and DNMT1, key DNA methylation maintenance factors, lowering the maintenance threshold and sensitizing cells to inhibition. They develop reporter assays driven by endogenous tumor‐suppressor gene promoters with enhanced sensitivity and dynamic range to demethylating drugs. This platform enables high‐throughput discovery of hypomethylating agents and optimization of epigenetic combination therapies.

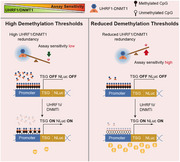

## 1 Introduction

Aberrant DNA hypermethylation at promoter CpG islands (CGIs) silences tumor suppressor genes (TSGs) and drives malignant progression of human cancers [1, 2, 3, 4]. These DNA methylation abnormalities are mitotically inherited through DNA methyltransferase 1 (DNMT1), whose recruitment to nascent DNA depends on the cofactor protein UHRF1 (ubiquitin like with PHD and RING finger domains 1) [5, 6, 7, 8]. Targeting either DNMT1's methyltransferase activity or UHRF1's chromatin‐reading functions holds therapeutic promise for cancer by reversing DNA hypermethylation‐mediated TSG silencing [9, 10, 11, 12, 13]. While nucleoside‐based DNMT inhibitors such as azacitidine (AZA) and decitabine (DAC) have validated this approach in hematologic malignancies [14, 15, 16, 17], their clinical efficacy has been hindered by dose‐limiting toxicities resulting from the formation of covalent DNA‐DNMT1 crosslinks and various non‐epigenetic effects [18, 19, 20]. Only partial, suboptimal DNA demethylation and TSG reactivation are achievable at patient‐tolerated doses [9, 21]. There remains an urgent need to develop novel DNA hypomethylating agents (HMAs) with distinct mechanisms of action.

Drug discovery for novel HMAs necessitates high‐throughput screening (HTS) assays with high specificity and sensitivity for UHRF1‐DNMT1 inhibition. The mainstays for this purpose are biochemical assays that monitor enzymatic methyl transfer or protein‐substrate binding, using purified protein fragments or domains [22, 23, 24, 25, 26, 27]. However, these assays fail to capture the dynamic, sequential process of UHRF1‐mediated DNMT1 targeting and other cofactor interactions that govern maintenance methylation in cells [7, 8, 28, 29]. Consequently, many biochemically active hits fail to translate into effective DNA demethylation in cellular contexts [10]. To address this, we and others have developed cell‐based reporter assays wherein reporter expression is repressed by hypermethylated promoters and reactivated upon demethylation [30, 31, 32, 33, 34, 35, 36, 37, 38]. When using endogenous promoters, these assays faithfully recapitulate the coordinated silencing of TSGs by epigenetic modifications, offering crucial insights into the clinical potential of HMAs and combination therapies [33, 37]. Nevertheless, we observed that a small amount of truncated, hypomorphic DNMT1 proteins can sustain most DNA methylation in HCT116 colorectal cancer (CRC) cells [39, 40, 41]. As a result, maximal reversal of promoter hypermethylation and TSG repression often requires reducing DNMT1 below an extremely low threshold [6], which limits the sensitivity of current cell‐based screening approaches for identifying compounds that robustly reactivate hypermethylated promoters.

In this study, we delineate the stringent threshold effects not only of DNMT1 but also of UHRF1 in maintaining cancer‐specific DNA methylation in CRC cells. We demonstrate that reduced redundancy of each protein can mitigate these thresholds, thereby sensitizing DNA methylation loss and TSG re‐expression to their respective inhibitors. These findings enable the development of recombinant reporter systems with substantially improved sensitivity and dynamic ranges for monitoring endogenous TSG reactivation through DNMT1 or UHRF1 inhibition. Using these platforms, we uncover potentially optimal dynamics of combining clinical‐stage histone modification inhibitors with DNMT1 or UHRF1 inhibitors for enhanced TSG reactivation. Our findings provide robust, sensitive tools for HTS of novel HMA and strategic insights for devising effective combination therapies for epigenetic cancer management.

## 2 Results

### 2.1 Development of a Luciferase‐Reporter Assay in DNMT1‐deficient Cells with Enhanced Sensitivity to DNMT1i‐Induced TSG Activation

**FIGURE 1:**
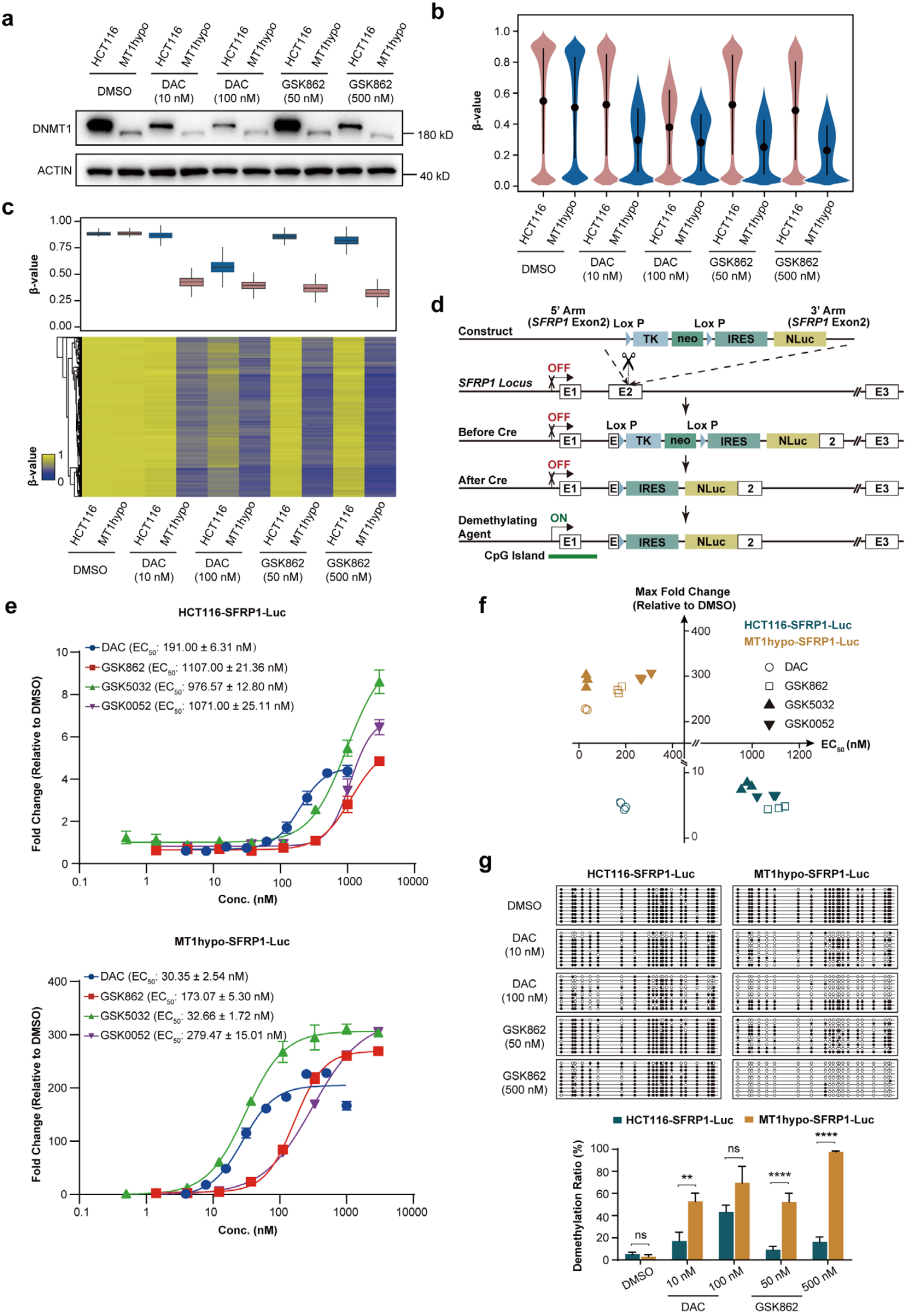
A recombinant luciferase reporter assay derived from MT1hypo cells demonstrates increased sensitivity and dynamic range in response to DNMT1 inhibition. (a) Western blot analysis of DNMT1 protein levels in HCT116 and MT1hypo cells treated with DMSO and the indicated compounds for 6 h. Data are representative of three independent experiments. (b) Violin plots showing global DNA methylation distributions in HCT116 and MT1hypo cells after 72‐h treatment with DMSO and the indicated compounds. DNA methylation levels are indicated by β‐value ranging from 0 to 1. (c) Promoter DNA demethylation analysis in HCT116 and MT1hypo cells following 72‐h treatment with DMSO and the indicated compounds. Hypermethylated cancer‐specific promoter probes with β‐value ≥ 0.75 in DMSO‐treated cells, as previously defined [11], were used to generate the boxplot and heatmap. (d) Schematic of SFRP1‐NLuc reporter construction in HCT116 and MT1hypo cells. A TK‐neo cassette, comprising a neomycin‐resistance gene (neo) driven by the thymidine kinase (TK) promoter, and an IRES‐NLuc cassette were inserted into exon 2 of *SFRP1* by CRISPR‐Cas9 mediated homologous recombination, with the scissor mark representing the Cas9 cut site. Transient Cre recombinase expression excised the LoxP‐flanked TK‐neo cassette, leaving NLuc under control of the endogenous *SFRP1* promoter. The CpG island is highlighted in green. (e) Dose‐responsive studies in MT1hypo‐SFRP1‐Luc and HCT116‐SFRP1‐Luc cells treated with the indicated DNMT1i for 72 h. Data are presented as mean ± SD (n = 3 technical replicates) and representative of three independent experiments. EC_50_ values are reported as mean ± SEM (n = 3 independent experiments). (f) Summary of EC_50_ values and maximum fold changes induced by the indicated compounds in MT1hypo‐SFRP1‐Luc and HCT116‐SFRP1‐Luc cells, derived from three independent experiments as depicted in (e). (g) (Top) Bisulfite sequencing‐based DNA methylation analysis of the *SFRP1* promoter region in reporter cells treated with the indicated compounds for 72 h. Open and filled circles indicate unmethylated and methylated CpG dinucleotides, respectively. (Bottom) Percentage of demethylation was calculated from all clones for each sample, represented as mean ± SEM (n = 10 clones). Statistical significance was determined by unpaired two‐tailed t‐test (**p < 0.01; ****p < 0.0001; and “ns” for not significant).

Genetic disruption of *DNMT1* in HCT116 cells produced severely hypomorphic alleles encoding a truncated DNMT1 protein, comprising approximately 10–15% of the wild‐type (WT) DNMT1 levels [6, 39, 40] (Figure 1a,b). These DNMT1 hypomorphic (MT1hypo) cells retained only about 5% residual DNMT enzymatic activity yet maintained the majority of genomic DNA methylation [6, 39, 40] (Figure 1b,c). Treatment with clinically relevant doses of DAC [9] or with the DNMT1‑selective, non‑nucleoside inhibitor GSK3484862 (GSK862) [42, 43], reduced both WT and truncated DNMT1 protein, resulting in significantly lower residual DNMT1 in MT1hypo compared to HCT116 cells (Figure 1a). This was accompanied by genome‐wide hypomethylation (Figure 1b; Figure S1a,b) and selective removal of cancer‐specific promoter hypermethylation at low inhibitor doses (10 nM DAC and 50 nM GSK862) in MT1hypo cells (Figure 1c). These observations support prior genetic depletion studies [6], underscoring a critical DNMT1 threshold for maintaining abnormal DNA methylation and suggesting that DNMT1 deficiency predisposes cells to DNMT1 inhibitors (DNMT1i)‐induced demethylation.

We engineered cell‐based reporters in both MT1hypo and parental HCT116 cells using a CRISPR‐Cas9 mediated homologous recombination approach [33, 44] to integrate the Nanoluciferase (NLuc) [45] gene into the endogenous *SFRP1* locus (Figure 1d; Figure S1c), a key WNT pathway antagonist primarily silenced by promoter hypermethylation in CRC tumors and HCT116 cells [46] (Figure S1d). This locus has previously been targeted for GFP reporter assay development in WT HCT116 cells with full DNMT1 expression [33]. Consistent with its role as a proxy for *SFRP1* expression, basal NLuc signals were essentially undetectable across recombinant clones but were robustly induced by DNMT1i in MT1hypo‐derived cells relative to DNMT1‐proficient cells (Figure S1e). The two most responsive clones (MT1hypo‐SFRP1‐Luc and HCT116‐SFRP1‐Luc) were selected for subsequent dose‐responsive studies. While DAC and the dicyanopyridine‐containing non‐nucleoside inhibitor GSK862, GSK3685032 (GSK5032) and GSK3830052 (GSK0052) [10] produced a maximum 4‐ to 9‐fold reporter activation in HCT116‐SFRP1‐Luc cells at concentrations of 1000–3000 nM (similar to DAC‐induced GFP fluorescence in previous HCT116‐SFRP1‐GFP assays [33]), comparable activation levels were achieved in MT1hypo‐SFRP1‐Luc at much lower concentrations (4.1–12.3 nM) (Figure S1f–i). At higher doses, these inhibitors elicited up to 200‐ to 300‐fold increases in NLuc signals in MT1hypo reporter cells. Consequently, EC_50_ values for DNMT1i were 4‐ to 30‐fold lower in DNMT1‐deficient reporter cells than in DNMT1‐proficient counterparts (Figure 1e,f), consistent with the more pronounced *SFRP1* promoter hypomethylation observed in MT1hypo reporters (Figure 1g; Figure S1j,k). Importantly, the robust DNMT1i‐induced reporter activation in MT1hypo‐SFRP1‐Luc cells was substantially blunted by ectopic expression of full‐length WT DNMT1 (Figure S1l,m). In contrast, the non‐nucleoside DNMT1i RG‐108 and SGI‐1027 failed to activate the reporter across tested concentrations, coinciding with their weak on‐target DNMT1 inhibitory effects [10] (Figure S1n,o). Overall, these data indicate that DNMT1 expression levels are functionally redundant for maintenance DNA methylation in CRC cells, and that reducing this redundancy dramatically increases the reporter's sensitivity to pharmacological DNMT1 inhibition.

### 2.2 Determining UHRF1 Thresholds in Maintaining DNA Hypermethylation‐Associated TSG Silencing in CRC Cells

**FIGURE 2:**
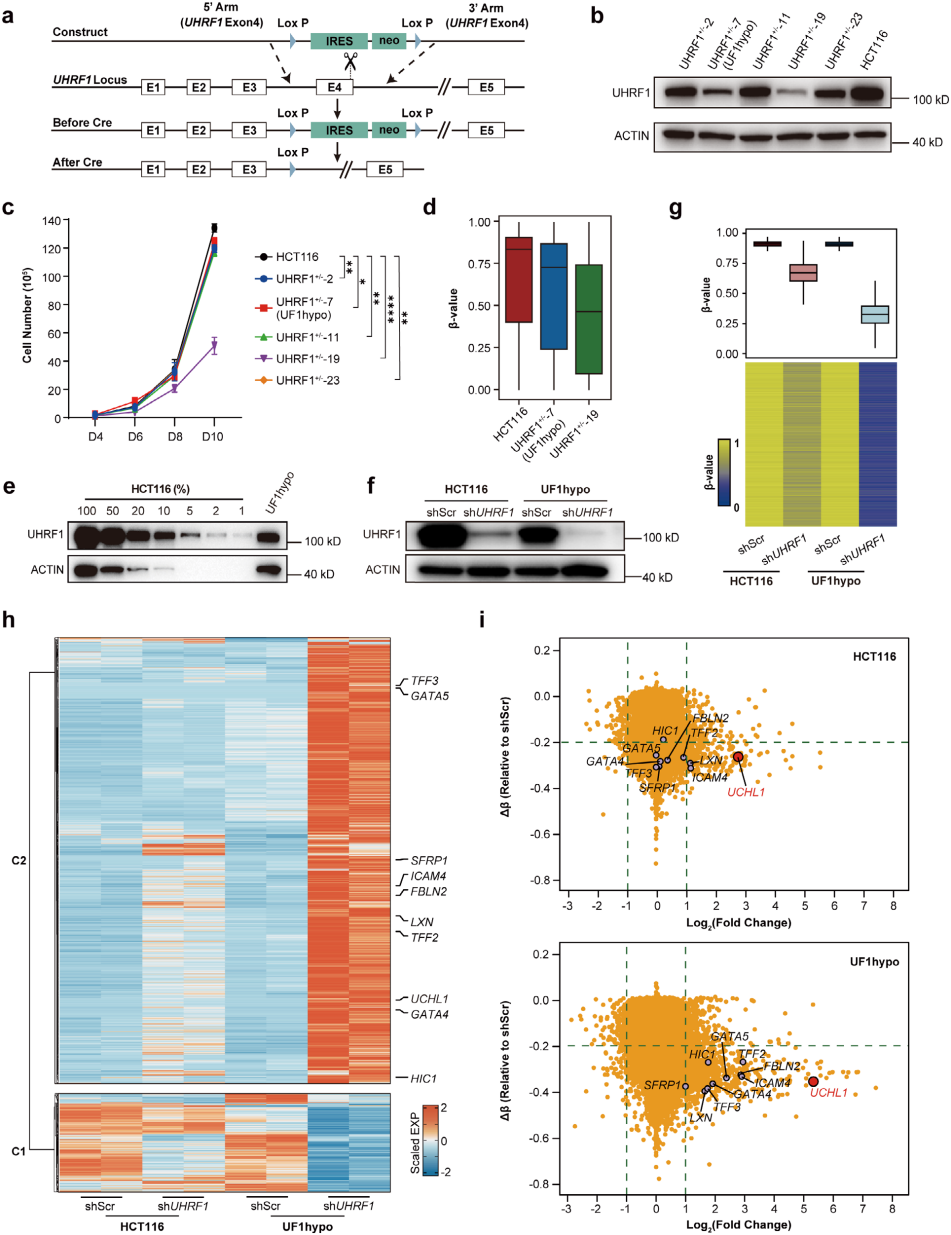
UHRF1 deficiency sensitizes DNA demethylation and TSG activation to UHRF1 inhibition. (a) Schematic illustrating UHRF1 knockout in HCT116 cells. Exon 4 of one *UHRF1* allele was replaced with an IRES‐neo cassette via CRISPR‐Cas9‐mediated homologous recombination, with the scissor mark indicating the Cas9 cut site. Transient Cre expression excised the IRES‐neo cassette, resulting in UHRF1 heterozygous knockout cells. (b) Western blot analysis of UHRF1 protein levels in WT HCT116 cells and five UHRF1 heterozygous knockout clones. (c) Cell growth curves of HCT116 and UHRF1 heterozygous knockout clones. Data are presented as mean ± SD (n = 3 technical replicates) and representative of three independent experiments. Statistical significance was determined by unpaired two‐tailed *t*‐test at day 10 (*p < 0.05, **p < 0.01, ****p < 0.0001). (d) Global DNA methylation levels (β‐value) in HCT116 cells and two UHRF1^+/−^ clones (UHRF1^+/−^‐7 and UHRF1^+/−^‐19). (e) Western blot analysis of UHRF1 protein levels in UF1hypo cells compared to serial dilutions of HCT116 cell extracts. (f) Western blot analysis of UHRF1 levels in HCT116 and UF1hypo cells with stable *UHRF1* knockdown. (g) Promoter DNA demethylation analysis in HCT116 and UF1hypo cells following stable *UHRF1* knockdown. Hypermethylated cancer‐specific promoter probes were used to generate the boxplot and heatmap. (h) Heatmaps showing the differentially expressed genes induced by *UHRF1* knockdown in UF1hypo and HCT116 cells, with key TSGs associated with promoter DNA hypermethylation labeled. (i) Scatter plots depicting the relationship between gene expression changes (x‐axis) and promoter methylation changes (y‐axis) in HCT116 (top) and UF1hypo cells (bottom) following *UHRF1* knockdown. Dashed vertical lines represent Log_2_(Fold Change) equal to −1 and 1, while horizontal lines denote Δβ values equal to −0.2. The representative TSGs in (h) were labeled, with *UCHL1* highlighted in red. Western blots in (b) and (e) are representative of three independent experiments, and the blot in (f) is representative of two independent experiments.

We investigated whether a similar threshold effect exists for UHRF1 in maintaining DNA methylation patterns in CRC cells. Utilizing a gene‐targeting approach [44], we excised exon 4 from one *UHRF1* allele in diploid HCT116 cells, resulting in heterozygous knockout clones with differentially reduced UHRF1 levels (Figure 2a,b; Figure S2a,b). While UHRF1^+/−^‐2, ‐11, and ‐23 clones exhibited approximate a 50% decrease in UHRF1 protein levels, a more pronounced reduction was observed in UHRF1^+/−^‐7 and UHRF1^+/−^‐19 cells (Figure 2b). This interclone variation might be due to differing degrees of allelic imbalance leading to unequal expression of the two *UHRF1* alleles within a single cell [47, 48], a phenomenon previously noted in *UHRF1* ablation studies [49]. Furthermore, while most UHRF1‐deficient clones displayed subtle‐to‐moderate reduction in growth compared to parental cells, UHRF1^+/−^‐19 cells, which had the lowest UHRF1 levels, exhibited substantially impaired proliferation (Figure 2c). This deep reduction in UHRF1 occurred with a markedly hypomethylated genome in UHRF1^+/−^‐19 cells (Figure 2d), in contrast to UHRF1^+/−^‐7 cells (designated UF1hypo), where roughly 20% residual UHRF1 maintained over 90% of overall DNA methylation (Figure 2d,e; Figure S2c). We further confirmed that UF1hypo cells did not exhibit compensatory changes in the protein levels of other major DNA methylation regulators, including DNMTs and DNA demethylases, similar to our prior observations in MT1hypo cells (Figure S2d) [6]. These data indicated that a key threshold level of UHRF1 is necessary and sufficient for maintaining CRC methylation, with UHRF1^+/−^‐19 cells falling below this threshold. Nevertheless, severe UHRF1 reduction compromised tumorigenic properties, an effect also observed in MT1hypo cells (Figure S2e,f), suggesting multifaceted roles of the UHRF1‐DNMT1 axis in supporting CRC growth.

We corroborated this threshold behavior by depleting UHRF1 in both HCT116 and UF1hypo cells (Figure 2f). The further, sharp reduction of remaining UHRF1 in UF1hypo cells led to more extensive loss of genomic (Figure S2g,h) and cancer‐specific DNA methylation (Figure S2i), including at cancer‐specific hypermethylated promoters maintained by key domains of UHRF1 [11, 12] (Figure 2g). Concomitantly, there was a dramatic re‐activation of a substantial number of key TSGs with abnormal promoter hypermethylation when extremely low levels of UHRF1 were achieved using shRNA knockdown in UF1hypo cells (Figure 2h,i). These encompassed antagonists of CRC proliferation (*UCHL1*, *LXN*, *SFRP1*) [11, 46], metastasis inhibitors (*ICAM4*, *FBLN2*) [11], positive regulators of intestinal epithelium differentiation (*GATA4*, *GATA5*, *TFF2* and *TFF3*) [50], and the DNA damage response activator *HIC1* [51, 52] (Figure 2i).

### 2.3 Establishing a Luciferase‐Reporter Assay for *UCHL1* De‐Repression in UF1hypo Cells with Enhanced Response to UHRF1 Inhibition

**FIGURE 3:**
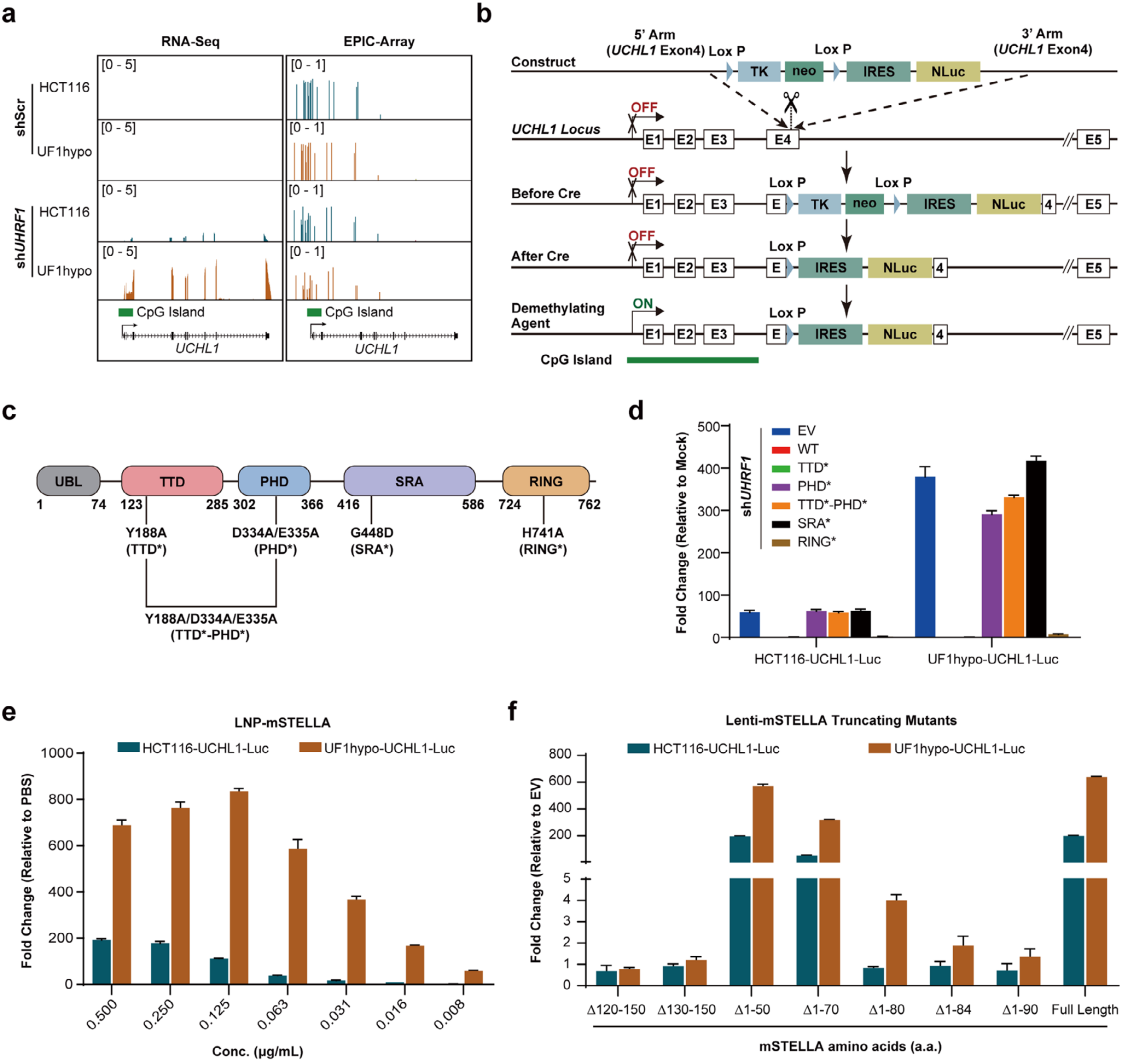
Establishing a UCHL1‐NLuc recombinant reporter assay in UF1hypo cells with improved responses to UHRF1 inhibition. (a) Transcriptome (RNA‐seq) and DNA methylome (EPIC‐Array) profiles at the *UCHL1* locus in HCT116 and UF1hypo cells following *UHRF1* knockdown. The CpG islands are highlighted in green. (b) Schematic of the UCHL1‐NLuc reporter construction in HCT116 and UF1hypo cells. CRISPR‐Cas9‐mediated homologous recombination inserted the TK‐neo and IRES‐NLuc cassettes into exon 4 of *UCHL1*, with the scissor mark indicating the Cas9 cleavage site. Transient Cre expression excised the TK‐neo cassette, leaving NLuc expression under the control of the endogenous *UCHL1* promoter. The CpG island is indicated in green. (c) Domain map of UHRF1 indicating the positions of point mutants disrupting individual or multiple domains. Domain boundaries are indicated by amino acid positions. (d) NLuc activity in HCT116‐UCHL1‐Luc and UF1hypo‐UCHL1‐Luc cells following endogenous UHRF1 depletion and complementation with the indicated domain mutants. Mock refers to cells co‐transduced with non‐silencing shRNA and an empty vector (EV) for ectopic UHRF1 expression. (e) NLuc activity in HCT116‐UCHL1‐Luc and UF1hypo‐UCHL1‐Luc cells treated with LNP‐mSTELLA for 72 h. (f) NLuc activity measured in HCT116‐UCHL1‐Luc and UF1hypo‐UCHL1‐Luc cells expressing either full‐length or truncated mSTELLA. NLuc activity data in (d–f) are presented as mean ± SD (n = 3 technical replicates) and representative of three independent experiments.

The threshold effect of UHRF1 in maintaining aberrant CRC methylation suggested that reducing UHRF1 expression could lower the barrier to TSG reactivation, thereby increasing sensitivity to UHRF1 inhibitors. Notably, *UCHL1*, a ubiquitin hydrolase whose reduced expression inversely correlates with promoter CGI hypermethylation (Figure S3a) and tracks with CRC progression [11], was more dramatically up‐regulated following promoter hypomethylation induced by *UHRF1* knockdown in UF1hypo cells (Figures 2i and 3a). We therefore generated UCHL1‐NLuc knock‐in alleles for monitoring endogenous *UCHL1* expression, distinct from previous luciferase reporters using in vitro methylated exogenous *UCHL1* promoter [38], and confirmed that UHRF1 abrogation induced substantially higher NLuc induction in UF1hypo‐derived clones compared to parental cells (Figure 3b; Figure S3b–d).

Disrupting UHRF1 domains responsible for histone H3 binding (PHD domain) or hemi‐methylated DNA recognition (SRA domain) phenocopied the loss of full‐length UHRF1 in reactivating silenced TSGs [11, 53, 54, 55]. We corroborated these findings through genetic complementation assays [11, 56, 57, 58], wherein endogenous UHRF1 was replaced with exogenous WT or domain mutants in the UF1hypo‐UCHL1‐Luc and HCT116‐UCHL1‐Luc reporter clones (Figure 3c; Figure S3e). Abolishing the PHD domain alone (PHD*), or combined inactivation with the H3K9me3‐binding TTD domain (TTD*‐PHD*), or the SRA domain (SRA*), resulted in reporter activation comparable to UHRF1 depletion (EV), with notably stronger effects in UF1hypo‐UCHL1‐Luc cells. Conversely, inactivating TTD alone (TTD*) produced minimal activation. Disruption of the E3 ligase activity of the RING domain caused only subtle changes in NLuc activity, aligning with its role in maintaining low‐density CpG methylation rather than dense CGI methylation at the *UCHL1* promoter [11, 53, 59] (Figure 3d).

Our recent work demonstrated that lipid nanoparticle (LNP)‐mediated mRNA delivery of mouse STELLA (mSTELLA), a natural UHRF1 inhibitor [60, 61], effectively reverses cancer‐specific DNA hypermethylation and tumorigenicity in CRC [12]. This activity requires a short region of mSTELLA that binds the TTD‐PHD module, disrupting UHRF1‐chromatin association [12, 60, 62]. Treatment with LNP‐mSTELLA de‐repressed NLuc signals dose‐dependently, with markedly greater sensitivity in UHRF1‐deficient cells, where 0.016 µg/mL of LNP‐mSTELLA elicited comparable reporter activation to 0.25 µg/mL in UHRF1‐proficient cells. Moreover, the response range was broader in UF1hypo‐UCHL1‐Luc cells (up to 835‐fold) compared to full UHRF1‐expressing cells (up to 192‐fold) (Figure 3e), and this expanded dynamic range was largely reversed by reintroduction of exogenous WT UHRF1 (Figure S3f,g). The heightened responsiveness in UF1hypo‐UCHL1‐Luc cells enabled detailed truncation analysis of mSTELLA to pinpoint the minimal region responsible for UHRF1 inhibition. Deletion of the extreme C‐terminus (Δ120–150 or Δ130–150) abolished mSTELLA activity, while mutants lacking substantial N‐terminal regions (Δ1–50 or Δ1–70) showed only modest reductions. Further N‐terminal deletions (Δ1–80, Δ1—84, and Δ1–90) caused rapid loss of function. Notably, residues 81–150 (Δ1–80 mutant) emerged as the minimal effective region in UHRF1‐deficient cells (Figure 3f).

In summary, reducing UHRF1 to levels that still supports promoter DNA hypermethylation, substantially lowers the threshold for effective UHRF1 inhibition, thus sensitizing cells for TSG reactivation as measured by our reporter assay.

### 2.4 Pilot HTS of a Bioactive Compound Library Revealed High Specificity of Reporter Assays to DNA Methylation Inhibition

**FIGURE 4:**
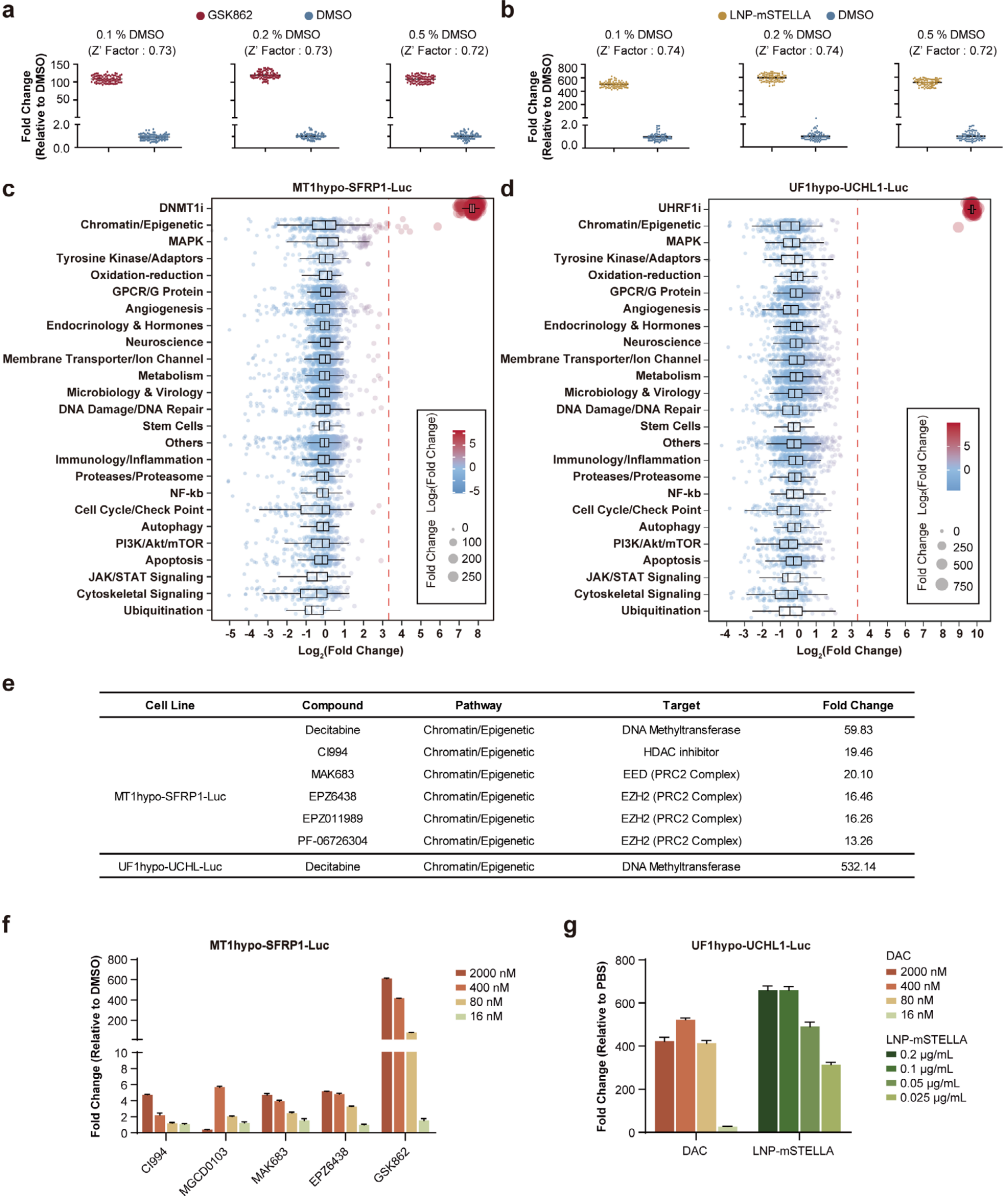
HTS of a Bioactive Compound Library using optimized reporter systems demonstrated high specificity for DNMT1i and UHRF1i. (a,b) Z’ factor analysis assessing the robustness of the optimized reporter systems in 384‐well plates under different DMSO concentrations. MT1hypo‐SFRP1‐Luc (a) and UF1hypo‐UCHL1‐Luc (b) cells were treated with GSK862 (500 nM) and LNP‐mSTELLA (0.25 µg/mL), respectively, for 72 h (n = 80 replicates per condition). (c,d) HTS of 7057 compounds in the Bioactive Compounds Library using MT1hypo‐SFRP1‐Luc (c) and UF1hypo‐UCHL1‐Luc assays (d) Screening was performed in single‐well format in 384‐well plates, with NLuc activity measured 72 h post‐treatment. NLuc activities of DNMT1i (2000 nM GSK862, GSK5032, GSK0052, or 200 nM DAC; n = 24 replicates per compound) and UHRF1i (0.5 µg/mL LNP‐mSTELLA; n = 48 replicates) in spike‐in plates were included. Scatterplots display the Log_2_(Fold Change) in NLuc activity (x‐axis) for each compound, categorized by target or signaling pathway modulation (y‐axis). Dot size and color represent activation levels. (e) Information of the hit compounds identified from the HTS screening using MT1hypo‐SFRP1‐Luc and UF1hypo‐UCHL1‐Luc assays. (f,g) Dose‐response validation of hit compounds using MT1hypo‐SFRP1‐Luc (f) and UF1hypo‐UCHL1‐Luc assays (g) performed in 96‐well plates after 72‐h treatment. LNP‐mSTELLA was included as a positive control for UF1hypo‐UCHL1‐Luc assay. Data are represented as mean ± SD (n = 3 technical replicates) and representative of three independent experiments.

Having established that the UHRF1‐DNMT1 axis's threshold effects in maintaining DNA hypermethylation‐associated TSG silencing can be leveraged to develop sensitive reporter assays, we assessed their applicability in high‐throughput compound screening. By miniaturizing both MT1hypo‐SFRP1‐Luc and UF1hypo‐UCHL1‐Luc assays into a 384‐well plate format, we first evaluated assay quality via the Z’ factor, a statistical metric reflecting signal dynamic range and data variability [63, 64]. Both assays showed strong, reproducible NLuc re‐activation following treatment with GSK862 and LNP‐mSTELLA, achieving Z’ factors above 0.7 across various DMSO concentrations, confirming their robustness and HTS suitability (Figure 4a,b).

Next, we conducted a pilot HTS using a Bioactive Compound Library containing over 7000 small molecules with well‐annotated targets and biological effects [65]. Spike‐in plates containing serially diluted DNMT1i (DAC, GSK862, GSK5032 and GSK0052) or the UHRF1i (LNP‐mSTELLA) were included every eight screening plates (Figure S4a) to ensure robust performance throughout the HTS process (Figure S4b,c). Consistent with the high sensitivity observed in 96‐well plates (Figures 1e and 3e), low doses of DNMT1i (8.2∼74.1 nM) or UHRF1i (0.008 µg/mL) significantly upregulated NLuc expression under screening conditions (Figure S4d,e). Using a tenfold increase in NLuc signal as the cutoff, the spiked DNMT1/UHRF1 inhibitors, and DAC from the library, elicited more pronounced NLuc activation than other compounds after 3 days of treatment (Figure 4c–e; Figure S4f,g, Tables S1 and S2). Additionally, treatment with the class I histone deacetylases (HDACs) inhibitor CI994 [66, 67], and inhibitors targeting EZH2 (EPZ6438 [68], EPZ011989 [69] and PF‐06726304 [70]) and EED (MAK683 [71]) within the PRC2 complex responsible for depositing repressive H3K27me3 marks, effectively reactivated NLuc in MT1hypo‐SFRP1‐Luc assays (Figure 4c,e). These findings were confirmed through dose‐response studies of CI994 and clinically relevant class I HDAC inhibitor (HDACi) MGCD0103, [72] along with two representative PRC2 inhibitors (Figure 4f). These histone modification inhibitors re‐expressed the silenced NLuc gene without altering *SFRP1* promoter DNA methylation (Figure S4h,i), supporting the idea that histone deacetylation and H3K27me3 cooperate with aberrant DNA methylation to reinforce *SFRP1* promoter silencing. Moreover, in screening conducted in UHRF1‐deficient reporter cells, only DAC among the library compounds induced significant NLuc reactivation (Figure 4d,e,g), accompanied by marked *UCHL1* promoter demethylation (Figure S4j,k). Collectively, these results demonstrate that our DNMT1‐ and UHRF1‐ hypomorphic reporter assays deliver high sensitivity and a pronounced specificity for compounds that induce DNA demethylation, validating their utility for identifying and prioritizing candidate DNA‐hypomethylating agents.

### 2.5 Sequential Inactivation of UHRF1/DNMT1 and Repressive Histone‐Modifying Enzymes Enhanced TSG Reporter Activation

**FIGURE 5:**
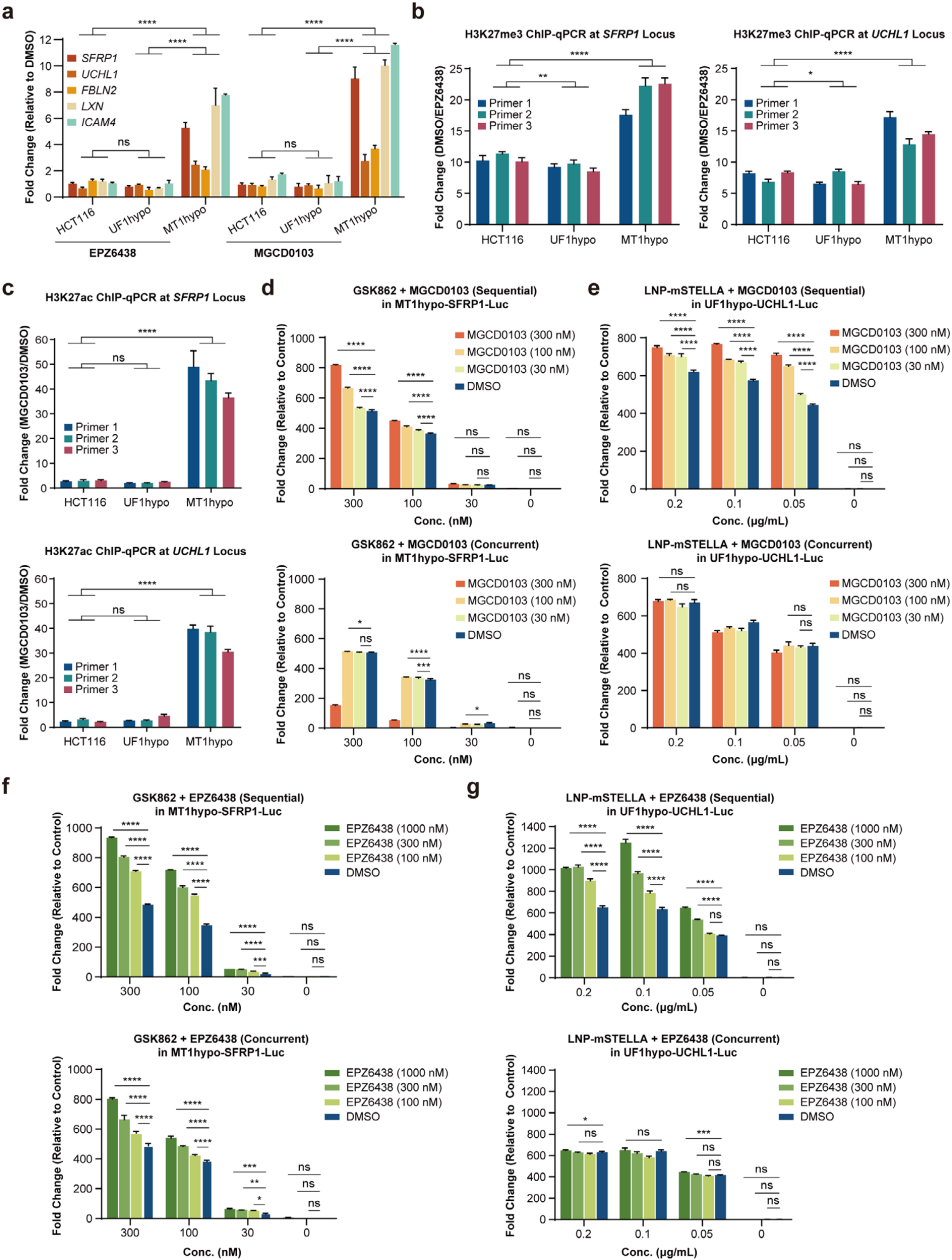
Sequential administration of DNMT1i or UHRF1i followed by EZH2i or HDACi induced more dramatic TSG reporter activation. (a) qPCR analysis of TSG expression in HCT116, UF1hypo, and MT1hypo treated with EPZ6438 (1000 nM) or MGCD0103 (300 nM) for 72 h. (b) H3K27me3 ChIP‐qPCR analysis at the *SFRP1* and *UCHL1* loci in HCT116, UF1hypo, and MT1hypo cells treated with EPZ6438 (1000 nM) for 72 h. The ratio of DMSO/EPZ6438 reflects the relative change in H3K27me3 levels. (c) H3K27ac ChIP‐qPCR analysis at the *SFRP1* and *UCHL1* loci in HCT116, UF1hypo, and MT1hypo cells treated with MGCD0103 (300 nM) for 72 h. The MGCD0103/DMSO ratio represents the relative change in H3K27ac levels. (d,e) NLuc activity in MT1hypo‐SFRP1‐Luc (d) and UF1hypo‐UCHL1‐Luc (e) after sequential or concurrent epigenetic inhibitor treatment. For sequential treatment, cells were pre‐treated with GSK862 or LNP‐mSTELLA for 3 days, followed by MGCD0103 for 2 days. In the concurrent regimen, cells received both agents simultaneously for 5 days. (f,g) NLuc activity in MT1hypo‐SFRP1‐Luc (f) and UF1hypo‐UCHL1‐Luc (g) cells following sequential or concurrent treatments with GSK862 or LNP‐mSTELLA combined with EPZ6438. Control indicates cells treated with equivalent volumes of DMSO or DMSO plus PBS (vehicle for LNP‑mSTELLA). qPCR data in (a) and NLuc activity data in (d‐g) are presented as mean ± SD (n = 3 technical replicates) and representative of three independent experiments. ChIP‐qPCR analysis in (b‐c) are presented as mean ± SD (n = 3 technical replicates) and representative of two independent experiments. Statistical significance was determined by two‐way ANOVA (*p < 0.05; **p < 0.01; ***p < 0.001; ****p < 0.0001; and “ns” for not significant).

We corroborated above findings by qRT‐PCR assays that both EZH2 inhibitor (EZH2i) and class I HDACi selectively de‐repressed *SFRP1* and other densely methylated TSGs in MT1hypo cells [11, 46] (Figure 5a). This contrasts with prior reports showing minimal reactivation of hypermethylated TSGs with EZH2i and HDACi alone in CRC cells with full DNMT1 expression [73, 74], despite their cooperative role with aberrant DNA methylation in TSG silencing [75, 76, 77, 78, 79]. We hypothesized that the very low expression of truncated DNMT1, lacking about 17 kDa of the N‐terminal region responsible for interactions with HDAC1/2 and EZH2 [29, 80, 81], may lead to an inefficient recruitment of these histone modifiers, thereby priming MT1hypo cells to be more susceptible to their inhibition. Indeed, EZH2i and HDACi treatments in MT1hypo cells induced a marked reduction in H3K27me3 levels and an increase in H3K27 acetylation (H3K27ac), a key active mark removed by class I HDACs (Figure 5b,c; Figure S5a–j). In contrast, these transcriptional and epigenetic changes were only marginal‐to‐moderate in UF1hypo and HCT116 cells (Figure 5a–c).

The aforementioned findings suggest that, although promoter CpG island demethylation is a primary driver of TSG reporter reactivation, the reporter readout can also reflect transcriptional de‐repression mediated by chromatin remodeling pathways that cooperate with aberrant DNA methylation to enforce stable silencing. Importantly, EZH2i‐ or HDACi‐induced reporter activation became readily detectable in both UF1hypo and MT1hypo cells when combined with DNMT1i or UHRF1i, which further reduced residual protein activity. Coinciding with previous studies [79, 82], sequential DNMT1i (3 days) followed by HDACi (2 days) elicited substantially greater TSG reporter activation than concurrent administration or single‐agent treatments over 5 days (Figure 5d; Figure S5k,l). A similar kinetic advantage was observed with sequential UHRF1i and HDACi (Figure 5e; Figure S5m). Interestingly, while concurrent DNMT1 and EZH2 inhibition amplified transcriptome changes in both CRC and immune cells [56, 83], sequential regimens of DNMT1i or UHRF1i followed by EZH2i yielded superior effects in de‐repressing TSG reporters (Figure 5f,g; Figure S5n–p). We further found that these sequential epigenetic treatments translated into more potent inhibition of CRC cell proliferation (Figure S6a,b). Moreover, consistent with our prior observations with DNMT1i [79, 83, 84], sequential administration of UHRF1i with HDACi or EZH2i induced stronger viral mimicry associated immune gene expression (Figure S6c,d), which may promote immune infiltration and strengthen anti‐tumor immune responses [85]. Collectively, these reporter systems provide a sensitive, quantitative platform to optimize the scheduling of DNA demethylating agents combined with histone modification inhibitors for more effective re‐expression of epigenetically silenced TSGs and improved cancer management.

## 3 Discussion

Developing DNA hypomethylating agents to restore the tumor‐suppressive functions of aberrantly hypermethylated TSGs represents a sought‐after cancer therapy paradigm [86, 87, 88, 89]. However, these drug discovery efforts are constrained by the requirement to reduce DNMT1 [6] or UHRF1 levels below a sharp threshold to achieve efficient DNA demethylation and TSG reactivation, limiting the sensitivity of drug screening and optimization. Here, we demonstrate that diminishing UHRF1/DNMT1 redundancy in maintenance methylation remarkably alleviates this deep threshold effect, thereby sensitizing cells to DNA methylation loss. We further established robust, cell‐based TSG reporter assays in UHRF1/DNMT1‐hypomorphic backgrounds. Compared with previously reported reporters driven by endogenous hypermethylated promoters [33, 37], our recombinant assays exhibited markedly improved sensitivity and dynamic range for detecting UHRF1 and DNMT1 inhibitors and for optimizing their use in epigenetic combination therapies.

Disrupting the maintenance methylation‐independent, scaffolding functions of the UHRF1‐DNMT1 axis likely accounts for the markedly lower demethylation threshold and the resulting enhanced sensitivity to pharmacologically induced DNA demethylation and TSG reactivation. Increasing recognition highlights the non‐catalytic, scaffolding roles of UHRF1 and DNMT1. Through their multi‐domain architectures, these proteins interact with key signal transducers to modulate lipid and glucose metabolism [90], and recruit additional repressors to assemble locus‐specific supramolecular complexes that regulate transcription and DNA damage repair [28, 49, 56, 91, 92, 93]. This is exemplified by the defective recruitment of the H3K4 demethylase LSD1 by hypomorphic DNMT1 in MT1hypo cells [91], leading to elevated H3K4me2 that may prime TSG induction by single‐agent HDACi [94] or EZH2i [95]. Our genetic depletion experiments suggest that a large fraction of UHRF1 and DNMT1 in HCT116 cells predominantly mediates these methylation‐independent functions. Removing these fractions leaves only small residual protein levels that remain sufficient to maintain bulk DNA methylation, yet markedly impair CRC tumor growth. Together, these findings point to an oncogenic role of the UHRF1‐DNMT1 axis beyond DNA methylation and motivate future studies to delineate the mechanistic basis of these scaffolding functions and their interplay with maintenance methylation in CRC progression.

Importantly, the antitumor efficacy of both nucleoside and non‐nucleoside DNMT1 inhibitors depend on a combination of enzymatic inhibition and DNMT1 protein depletion [10, 96, 97, 98, 99]. Compared with other cellular readouts, our DNMT1‐deficient MT1hypo‐derived reporter system can more unambiguously disentangle the DNA demethylating activity of compounds from their effects on DNMT1's structural, scaffolding roles, a distinction that has recently aided development of DNMT1/HDAC dual inhibitors [78]. Recent work also highlights non‐canonical functions of UHRF1 in regulating DNA methylation homeostasis in CRC, partly by prompting the *de novo* activities of DNMT3A/3B via the TTD domain [55]. Although inactivating TTD mutations have minimal effect on maintenance methylation [11, 100] or TSG reporter activation, they significantly impair UHRF1's interaction with and stimulation of DNMT3A/3B [55]. Rationally designed, mSTELLA‐mimetic UHRF1 inhibitors that selectively target the PHD domain [101] and the linked TTD‐PHD domains [12] could thus facilitate the dissection of UHRF1's canonical, DNMT1‐dependent functions versus its non‐canonical roles and inform rational therapeutic strategies. The UF1hypo reporter assays, which respond robustly to disruption of the PHD and TTD‐PHD domains, provide a practical platform to advance these efforts. Moreover, these mSTELLA mimetics could serve as warheads for developing targeted UHRF1 degraders [102], thereby concurrently abrogating both methylation‐dependent and methylation‐independent oncogenic functions.

The high sensitivity of our TSG reporter assays may also be attributed to the selection of TSG targets for in situ modification. *SFRP1* and *UCHL1* are frequently silenced by promoter CGI hypermethylation in CRC, and their loss contributes to tumorigenesis and poor prognosis [11, 46]. Genetic or pharmacological abrogation of DNMT1 effectively de‐repressed *SFRP1* [73], motivating our earlier development of a GFP reporter to monitor endogenous *SFRP1* in WT HCT116 cells [33]. Similarly, *UCHL1* exhibited the largest transcriptional induction among a panel of hypermethylation‐silenced TSGs following UHRF1 depletion, despite only moderate promoter demethylation. Finally, using NLuc as the reporter, which yields > 100‐fold greater luminescence and lower background than conventional luciferases [103], further improved assay sensitivity and signal‐to‐noise ratio for detecting TSG re‐expression induced by UHRF1 or DNMT1 inhibition.

The extensive crosstalk between DNA methylation and histone modifications underpins combined epigenetic cancer therapy [104, 105, 106, 107]. Our reporter assays not only recapitulated the superior TSG reactivation achieved by sequential DNMT1i or UHRF1i followed by HDACi, an approach with demonstrated preclinical efficacy, consistent with our current CRC findings, and under ongoing clinical evaluation [79, 82, 108], but also unveiled previously underappreciated kinetic advantages for sequential administration of DNA demethylating agents followed by EZH2 inhibition [109, 110]. After promoter CGI hypomethylation, re‐expressed TSGs often acquire a bivalent chromatin configuration, marked by co‐occurrence of the active mark H3K4me3 and the repressive mark H3K27me3 [94]. This configuration maintains genes in a poised, low‐transcription state in embryonic stem cells and permits rapid activation upon loss of H3K27me3 during differentiation [111, 112]. However, such a stem‐like bivalent state can limit full re‐activation of silenced TSGs post‐HMA treatment, as compensatory H3K27me3 may re‐establish repression [83]. Our data suggest that a short DNA hypomethylation priming period (3 days in this study) might stabilize a bivalent chromatin state that increases dependence on EZH2 activity. Consequently, sequential DNMT1i or UHRF1i followed by EZH2i more effectively blocks the compensatory H3K27me3 switch and potentiates TSG induction relative to concurrent treatment. Unlike previous reporter systems driven by exogenous promoters [110], our recombinant assays employing endogenous TSG promoters more accurately captures the dynamic, sequential emergence of bivalent states in response to DNA hypomethylation, thereby providing mechanistic insight with improved translational relevance, as observed in CRC cells with intact DNMT1 and UHRF1 expression. Further preclinical and clinical studies are needed to evaluate the therapeutic implications of sequentially inhibiting DNA and H3K27 methylation in both cancer cells and T cells, the latter potentially enabling adoptive cell therapy via reprogramming T cells into NK‐like cells with enhanced antitumor activity [56].

Moreover, the translational significance of these sequential epigenetic regimens is supported by the stronger induction of viral‐mimicry associated immune responses with the newly developed UHRF1i‐based combinations. Such immune activation may remodel the tumor microenvironment and enhance responses to immune‐checkpoint blockade, an approach under active investigation for DNMT1i‐based therapies in solid tumors [79, 113]. Future studies are therefore warranted to evaluate therapeutic paradigms combining UHRF1i with histone‐modifying agents and immune‐checkpoint inhibitors.

In summary, our study demonstrates that the threshold effects in maintenance DNA methylation in cancer can be exploited to develop robust, high‐throughput reporter assays with superior sensitivity to UHRF1 and DNMT1 inhibition. These findings provide valuable tools for discovering and optimizing next‐generation UHRF1 or DNMT1 inhibitors, and for devising new, optimal combination strategies with other epigenetic therapies to improve cancer treatment.

## 4 Experimental Section

### 4.1 Cell Culture

HCT116 and the isogenic DNMT1‐ or UHRF1‐hypomorphic cell lines were cultured in McCoy's 5A medium supplemented with 10% fetal bovine serum (FBS; NEWZERUM) and maintained at 37°C in a humidified 5% CO_2_ atmosphere. Cells were periodically tested for mycoplasma (Vazyme) and remained negative.

### 4.2 Chemicals

The following compounds were obtained from MedChemExpress (MCE): GSK3484862 (HY‐135146), GSK3685032 (HY‐139664), RG‐108 (HY‐13642), and SGI‐1027 (HY‐13962). The following compounds were obtained from TargetMol: EPZ6438 (T1788), MGCD0103 (T2512), CI994 (T1888), MAK683 (T15201), EPZ011989 (T2435), and PF‐06726304 (T12428L). Decitabine was purchased from Sigma‐Aldrich (A3656). GSK3830052 was synthesized according to the method described in patent WO2017216727A1 from commercially available reagents.

### 4.3 Nanoluciferase (NLuc) Activity Assay

Cells were seeded in 96‐well plates at 4500 cells per well and incubated for 16–18 h prior to compound addition. After 72 h of treatment with the indicated compounds, Nanoluciferase (NLuc) activity was measured using the Nano‐Glo Luciferase Assay System (Promega) according to the manufacturer's instructions. Luminescence was recorded with a GloMax Discover microplate reader (Promega).

### 4.4 Plasmid Construction

Short hairpin RNA (shRNA) sequences targeting *UHRF1* were cloned into pLKO.1 (Addgene, 10878). Single guide RNAs (sgRNAs) were designed with the Benchling CRISPR Guide RNA Design tool (https://www.benchling.com/crispr) and subcloned into pX330 (Addgene, 110403). shRNA and sgRNA primer sequences are listed in Table S3. For knock‐in plasmid construction, homology arms (800–1000 bp) flanking the Cas9 cleavage sites at the *SFRP1* and *UCHL1* loci were PCR amplified from HCT116 genomic DNA and cloned into our previously described donor plasmid used to generate the recombinant SFRP1‐GFP allele [33], with GFP replaced by Nanoluciferase (NLuc) in the current construct. Homology arms for *UHRF1* were PCR amplified and cloned into a donor plasmid containing an internal ribosome entry site (IRES) upstream of a neomycin‐resistance gene (neo) [44]. This IRES‐neo cassette is flanked by Lox P sites, facilitating its excision by Cre recombinase.

cDNAs encoding mSTELLA truncation mutants were PCR amplified and subsequently cloned into pLenti‐III‐EF1α (Applied Biological Materials, LV043) by restriction‐enzyme digestion and ligation. Wild‐type (WT) UHRF1 and its point mutants were cloned into pLenti6m‐STAP as previously described [11].

### 4.5 Construction of Recombinant Reporter Systems

Cells were seeded in 6‐well plates at 3 × 10^5^ cells per well. After 16–18 h, cells were co‐transfected with 2 µg pX330 carrying the sgRNA and 1 µg donor plasmid using PEI 40000 (Yeasen). 48 h after transfection, cells were transferred to 10‐cm dishes. G418 selection (InvivoGen) at 400 µg/mL was initiated 12 h later and maintained for 5 days. To generate UHRF1 heterozygous knockout clones, G418‐selected cells were single‐cell cloned by limiting dilution in 96‐well plates. Following PCR genotyping and functional validation, the selected UHRF1^+/−^ clone was infected with adenovirus expressing Cre recombinase (Ad‐Cre) and single‐cell cloned to isolate IRES‐neo excised lines. For recombinant reporter construction, G418‐selected pools were treated with Ad‐Cre and individual clones were screened for proper recombinants by PCR. Primer sequences used for genotyping are listed in Table S4.

### 4.6 Lentivirus Production and Stable Cell Line Generation

Target plasmids were co‐transfected with packaging plasmids (psPAX2 and PMD2.G) into HEK293T cells to produce lentivirus. Medium was replaced 8 h after transfection and viral supernatants were collected at 24, 48 and 72 h after the medium change. Supernatants were clarified by centrifugation at 1000 rpm for 5 min, filtered through a 0.45 µm membrane, and concentrated by PEG8000 (Sigma‐Aldrich). Viral particles were pelleted by centrifugation at 4000 × g for 20 min and resuspended in PBS.

To establish stable cell lines expressing mSTELLA (or mutants) or to deplete UHRF1, target cells were seeded in 6‐well plates overnight and incubated with virus for 48 h. Infected cells were transferred to 10‐cm dishes and selected with 2 µg/mL puromycin (InvivoGen) for 48 h. Stable pools were subsequently maintained in 1 µg/mL puromycin, and cells were harvested on day 8 or day 12 post‐infection for downstream experiments.

### 4.7 Genetic Complementation Assay

The genetic complementation assay was performed as previously described [11]. Briefly, HCT116‐UCHL1‐Luc and UF1hypo‐UCHL1‐Luc cells were co‐transduced with lentiviruses encoding WT or mutant UHRF1 together with an shRNA targeting endogenous UHRF1 for 48 h. Cells were then transferred to 10‐cm dishes, and 24 h later selection was initiated with blasticidin (10 µg/mL) and puromycin (2 µg/mL) (InvivoGen). Dual‐antibiotic selected stable populations were harvested at day 12 post‐infection for downstream experiments.

### 4.8 Western Blot

Cell pellets were collected and washed twice with ice‐cold PBS, then lysed in 4% SDS buffer. Lysates were clarified using a homogenizer spin column (Omega), and protein concentrations were measured with the Pierce BCA Protein Assay Kit (Thermo Fisher Scientific). Equal amounts of protein were separated by 10% SDS‐PAGE and transferred onto PVDF membranes. Membranes were blocked with 5% milk in TBST for 1 h at room temperature and incubated overnight at 4°C with primary antibodies: anti‐UHRF1 (Sigma‐Aldrich, MABE308), anti‐DNMT1 (Sigma‐Aldrich, D4692 or Abcam, ab92314), anti‐H3K27me3 (Cell Signaling Technology, 9733S), anti‐H3K27ac (Active Motif, 39133), anti‐H3 (Abcam, ab1791), and anti‐β‐Actin (Sigma‐Aldrich, A5441), anti‐DNMT3A (Novusbio, NB120‐13888), anti‐DNMT3B (Cell Signaling Technology, 67259S), anti‐TET2 (Sigma, MABE462) and anti‐TET3 (GeneTex, GTX121453). Following washing with TBST, membranes were incubated with HRP‐conjugated secondary antibodies for 1 h at room temperature. Protein bands were detected using enhanced chemiluminescence reagents (Thermo Fisher Scientific).

### 4.9 Cell Proliferation, Colony Formation and Soft Agar Colony Formation Assays

HCT116 cells and UHRF1^+/−^ clones (#2, #7, #11, #19 and #23) were seeded in 96‐well plates at 2 × 10^4^ cells per well (day 0) and cultured under standard conditions. During passaging, cells were transferred to progressively larger vessels: 24‐well plates on day 2, 6‐well plates on day 4, 6‐cm dishes on day 6, and 10‐cm dishes on day 8. Cell counts were performed on days 4, 6, 8, and 10.

For colony formation assays, HCT116 cells were first treated with DNA methylation inhibitors (GSK862 or LNP‐mSTELLA) for 72 h. The medium was then replaced with fresh medium containing histone modification inhibitors (EPZ6438 or MGCD0103) for an additional 48 h. After sequential treatment, 5000 viable cells were seeded into 6‐well plates. After 7 days, colonies were fixed with 4% formaldehyde for 15 min and stained with 0.5% crystal violet for 30 min. Plates were washed, air‐dried, scanned, and colony images were recorded. For soft agar assays, 6‐well plates were pre‐coated with a 0.6% agarose base layer, followed by a 0.4% agarose middle layer. Next, 5000 viable HCT116, MT1hypo, or UF1hypo cells were resuspended in a 0.4% agarose top layer and cultured for 2–3 weeks. Colonies were fixed with 4% paraformaldehyde and stained with 1 µg/mL ethidium bromide for visualization. Images were acquired, and colony numbers were quantified using ImageJ.

### 4.10 Real‐Time qPCR

Cell pellets were lysed with RNAiso Plus (Takara), then mixed with chloroform. After centrifugation at 4°C, the supernatant was combined with ice‐cold isopropanol and incubated on ice for 10 min. The RNA pellet was washed twice with 75% ethanol, then dissolved in nuclease‐free water. RNA concentration and purity were assessed using a NanoPhotometer (IMPLEN). For cDNA synthesis, 1 µg of total RNA was reverse transcribed with PrimeScript RT Master Mix (Takara). Quantitative PCR (qPCR) analysis of tumor suppressor genes (*SFRP1*, *UCHL1*, *FBLN2*, *LXN* and *ICAM4*), ERVs (*ERVFXA34*, *ERV‐Fc1*, *envT, ERV‐FRD 1* and *LINE‐1*) and immune‐related genes (*TLR3*, *RIG‐I*, *IFI44*, *MDA5*, *IFI27*, *IRF3*, *CT45A1 and HLA‐B*), were performed using the SYBR Green PCR Kit (Takara). Fold changes were calculated via the ΔΔCt method, with β‐actin as the internal control and DMSO as the calibrator. Primer sequences are listed in Table S5.

### 4.11 mRNA Synthesis

mRNA was synthesized in vitro via T7 RNA polymerase‐mediated transcription using a linear DNA template encoding the target protein. The reaction incorporated standard NTPs (ATP, CTP, GTP) and N1‐methylpseudouridine‐5'‐triphosphate (N1‐Me‐pUTP) as a UTP substitute. Co‐transcriptional capping incorporated the cap analog into the 5’ end, and E. coli poly(A) polymerase added a poly(A) tail at the 3' end. RNA quality was evaluated by examining RNA length, poly(A) tail length, purity, and other relevant parameters.

### 4.12 Lipid Nanoparticle Formulation

LNPs were prepared by microfluidic mixing as previously described [12, 114]. Briefly, the ionizable lipid, DSPC, cholesterol, and DMG‐PEG2000 were dissolved in ethanol at a molar ratio of 48.5:10:40:1.5. This lipid solution was rapidly mixed with an aqueous phase containing mRNA in 50 mM sodium citrate buffer (pH 5.0) at a volume ratio of 1:3 using the microfluidic mixer (Inano E, Micro&Nano). The LNP formulation was dialyzed against PBS (pH 7.2) for at least 18 h using dialysis cassettes (Thermo Scientific). After dialysis, the formulation was diluted with PBS, filtered through a 0.22 µm pore size membrane filter, and stored at 4°C.

### 4.13 Bisulfite Sequencing PCR

Genomic DNA was extracted using the Wizard Genomic DNA Purification Kit (Promega) following the manufacturer's instructions. Bisulfite conversion was carried out using the EZ DNA Methylation‐Lightning Kit (ZYMO RESEARCH) with 500 ng of genomic DNA. The converted DNA served as a template for PCR amplification of target regions in the *SFRP1* and *UCHL1* genes. PCR products were purified using the QIAquick Gel Extraction Kit (QIAGEN). The purified fragments were cloned into the pClone007 vector (TSINGKE) for Sanger sequencing. Primer sequences for BSP are listed in Table S6.

### 4.14 Chromatin Immunoprecipitation‐qPCR

ChIP was performed as previously described [11]. Briefly, cells were crosslinked with 1% formaldehyde for 10 min at room temperature and quenched with 0.125 M glycine. Cells were collected and washed twice with ice‐cold PBS. Chromatin was sequentially extracted and lysed using: (1) CEBN buffer (10 mM HEPES [pH 7.8], 10 mM KCl, 1.5 mM MgCl_2_, 0.34 M sucrose, 10% glycerol, 0.2% NP‐40), (2) CEB buffer (CEBN without NP‐40), and (3) nuclei lysis buffer (50 mM Tris‐HCl [pH 8.1], 10 mM EDTA [pH 8.0], 1% SDS). Chromatin was sonicated to fragments of 200–1000 bp. For immunoprecipitation, 50 µg of chromatin was incubated overnight at 4°C with 5 µg target‐specific antibody (or IgG control). Antibody‐chromatin complexes were captured with pre‐blocked Protein A/G magnetic beads (Thermo Fisher) for 3 h at 4°C with gentle rotation. Beads were washed sequentially with low salt buffer (20 mM Tris‐HCl [pH 8.0], 150 mM NaCl, 2 mM EDTA, 0.1% SDS, 1% Triton X‐100), high salt buffer (20 mM Tris‐HCl [pH 8.0], 500 mM NaCl, 2 mM EDTA, 0.1% SDS, 1% Triton X‐100), LiCl buffer (10 mM Tris‐HCl [pH 8.0], 250 mM LiCl, 1% NP‐40, 1% deoxycholic acid, 1 mM EDTA) and twice with TE buffer. Bound complexes were eluted and reverse crosslinked by overnight incubation at 65°C in 0.3 M NaCl, followed by RNase A and Proteinase K treatments. DNA was purified with the QIAquick PCR Purification Kit (QIAGEN) and analyzed by qPCR to assess enrichment of target regions. All buffers contained protease and phosphatase inhibitors, and samples were kept at 4°C or on ice unless otherwise specified. Primer sequences are listed in Table S7.

### 4.15 Z′ Factor and Z Factor Evaluation

Cells were seeded at 1000 cells per well in 384‐well plates. After 16–18 h, cells were treated with the indicated positive and negative control compounds for 72 h. NLuc activity was then measured. The key statistical parameters, including means (µp, µn) and standard deviations (σp, σn) of positive and negative controls, were used to calculate the Z’ and Z factors according to equation: Z’ or Z = 1 − [3(σp + σn) / |µp − µn|] [63].

### 4.16 High‐Throughput Screening

Cells were seeded in 384‐well plates at 1000 cells per well using a Multidrop Combi dispenser (Thermo Fisher) and cultured overnight. The next day, compounds from source plates (10 mM stocks in DMSO, stored at −80°C) from the Bioactive Compound Library were delivered at 10 nL per well using the PerkinElmer Explorer G3 HTS system, followed by 72 h of incubation. NLuc activity was measured with a PerkinElmer EnVision plate reader. For quality control, three spike‐in plates were incorporated into the HTS workflow, with one spike‐in plate analyzed after every eight test plates to monitor assay consistency.

### 4.17 DNA Methylation Analysis

Genome‐wide DNA methylation profiles were determined using Illumina's Infinium MethylationEPIC BeadChip v1.0 microarray. Raw data files were processed with the R package ChAMP v2.26.0 [115], which calculated methylated (M) and unmethylated (U) signal intensities for each probe, along with detection p‐values. β‐values were calculated as M/(M+U), representing the fraction methylated at each probe. Probes with a detection p‐value > 0.01 were excluded from analysis. Probe annotations were obtained using the IlluminaHumanMethylationEPICanno.ilm10b4.hg19 package v0.6.0 in R, and loci were converted to GRCh38 coordinates via UCSC LiftOver to facilitate multi‐omics analysis and annotation of promoter and gene body related probes. Cancer‐specific and enhancer probes were selected based on prior studies [11, 12]. Visualization of methylation levels at specific loci was performed using the IGV v2.16× genome browser [116], and methylation data in relevant probes were visualized with the ComplexHeatmap v2.12.1 package in R [117].

### 4.18 RNA‐Seq Data Analysis

Potential adapters and low‐quality reads were removed using Fastp v0.21.0 [118]. The cleaned reads were mapped to the human GRCh38 reference genome using HISAT2 v2.0.4 [119]. Gene counts and TPM (transcripts per kilobase million) expression levels were quantified with StringTie v2.1.4 [120]. Protein‐coding genes annotated by GENCODE (GRCh38) were retained for downstream analysis, and only genes with a maximum TPM > 1 in any sample of each comparison group were included. Differentially expressed genes (DEGs) were identified using the DESeq2 package v4.3 in R, applying thresholds of |Log_2_(Fold Change)| > 1 and adjusted p‐value < 0.05. Visualization and exploration of specific gene loci were conducted with the IGV v2.16× genome browser [116]. DEG expression patterns were visualized using the ComplexHeatmap v2.12.1 package in R [117].

### 4.19 TCGA‐COAD Data Analysis

Transcriptomic and DNA methylation data for colon adenocarcinoma (COAD) from TCGA were obtained using TCGAbiolinks (v2.24.3) in R, including RNA‐seq gene expression, methylation β‐values from Illumina HumanMethylation450 arrays, and clinical annotations. Expression matrices were derived from TPM values, focusing on primary tumor samples (TCGA barcode ending in 01A) and adjacent normal tissues (barcode ending in 11A). CpG probes within ± 1500 bp of transcription start sites (TSS, GRCh38) were averaged per gene, and genes lacking promoter coverage were excluded. Analyses paired gene expression with methylation profiles based on sample barcode and gene symbol. Promoter methylation and expression correlations were evaluated with linear regression: Log_10_(TPM+1) ∼ β‐value, and significance was assessed by Pearson's r and p‐value.

### 4.20 In Vivo Xenograft Study

Animal experiments were approved by the Institutional Animal Care and Use Committee (IACUC, No. 2024039) of the Guangzhou Institutes of Biomedicine and Health (GIBH) and were conducted in accordance with relevant ethical regulations. To align with the patient's gender, 5‐ to 6‐week‐old male NOD‐SCID mice were used. For subcutaneous xenografts, 1 × 10^6^ viable HCT116, MT1hypo, or UF1hypo cells were re‐suspended in 0.1 mL of a 1:1 (v/v) mixture of PBS and Matrigel and injected into the flank of each mouse. Tumor dimensions were measured every 2 days using digital calipers (length [L], width [W], and height [H]), and tumor volume (V) was calculated as V = 0.5 × (L × W × H). Body weight and general condition were monitored throughout the study. Mice were euthanized when tumor volume approached the protocol‐specified maximum (2000 mm^3^), when body weight loss exceeded 20%, or when distress signs were observed.

### 4.21 Ethics Approval Statement

This study was conducted in accordance with the ethical regulations of GIBH, Chinese Academy of Sciences. All animal experimental protocols were approved by the GIBH Institutional Animal Care and Use Committee (IACUC, No. 2024039) and performed following institutional guidelines.

### 4.22 Statistical Analysis

Unless otherwise stated, data are presented as mean ± SEM for biological replicates or mean ±SD for technical replicates, as specified in the figure legends. Quantitative assays, including NLuc activity, cell proliferation, qPCR, and soft agar colony formation, are presented as mean ± SD from three technical replicates and representative of three independent experiments. All other data are representative of three independent experiments, except that the immunoblot in Figure 2f and the ChIP‐qPCR (Figure 5b,c and S5c–e,h–j) are representative of two independent experiments.

Statistical analyses were performed using GraphPad Prism (https://www.graphpad.com/). Specifically, the *SFRP1* promoter methylation levels (Figure 1g), cell proliferation (Figure 2c), soft agar colony formation (Figure S2e), and in vivo tumor xenograft growth (Figure S2f) were analyzed using an unpaired two‐tailed t‐test. Data from NLuc activity assays (Figure 5d–g and S1e, S3d, S5k,n), qPCR (Figure 5a), and ChIP‐qPCR (Figure 5b,c) were analyzed using two‐way ANOVA. For bioinformatics analyses, including genome‐wide DNA methylation and gene expression profiling, were conducted in R (http://www.r‐project.org). Wilcoxon rank‐sum tests were employed to assess differences in DNA methylation and gene expression. For correlation analyses (Figures S1d and S3a), *p*‐values for Pearson's correlation coefficients were calculated using unpaired two‐sided t‐tests within linear regression models. *p*‐values < 0.05 were considered statistically significant. Demethylation ratios across *SFRP1* promoter CpG sites (Figure S1l) were analyzed using a paired two‐tailed t‐test. Significance levels were indicated by asterisks: *p < 0.05, **p < 0.01, ***p < 0.001, ****p < 0.0001, and “ns” for not significant.

## Author Contributions

C.X. and X.K. conceived and designed the research; C.X. performed the experiments and data analysis; JI.W. assisted with the bioinformatics analyses; D.W. prepared the LNP formulations; Y.C. and W.B. optimized the protocol and provided technical advice; H.G. assisted with the analysis of ChIP‐qPCR data; YA.C. helped with high‐throughput screening. X.K., C.X., Z.G. and S.B.B. wrote the manuscript. S.B.R., J. H., J.W., L.Z., X.C. and B.Q. supplied important research reagents and technical advice.

## Funding

This work was supported by the R&D Program of Guangzhou Laboratory (GZNL2023A02003), National Natural Science Foundation of China (22277010, 92353303, and 22477013), the Major Research Project of Guangzhou Institutes of Biomedicine and Health, Chinese Academy of Sciences (GIBHMRP2025‐02), and the Strategic Priority Research Program of the Chinese Academy of Sciences (XDB1250000).

## Conflicts of Interest

The authors declare no conflicts of interest.

## Materials & Correspondence

Further information and requests for resources and reagents should be directed to and will be fulfilled by the Lead Contact, Xiangqian Kong (kong_xiangqian@gibh.ac.cn) and/or Stephen B. Baylin (sbaylin@jhmi.edu).

## Supporting information




**Supporting File 1**: advs74964‐sup‐0001‐SuppMat.pdf.

**Supporting File 2**: advs74964‐sup‐0002‐TableS1.xlsx.

**Supporting File 3**: advs74964‐sup‐0003‐TableS2.xlsx.

## Data Availability

DNA methylation and gene expression data generated in this study have been deposited in the Gene Expression Omnibus (GEO) database under accession codes GSE305299and GSE305298.
