# Supplementary material for: Reduced Maintenance DNA Methylation Thresholds Enable Sensitive Reporter Assays for UHRF1 and DNMT1 Inhibition
Source: Adv Sci (Weinh). 2026 Mar 26;13(32):e19080. doi: 10.1002/advs.202519080 (PMC13252609; doi:10.1002/advs.202519080)
Supplement: Supplementary file 1 — Supporting File 1: advs74964‐sup‐0001‐SuppMat.pdf. [file ADVS-13-e19080-s001.pdf]

Supplementary Information for

Reduced Maintenance DNA Methylation Thresholds Enable Sensitive Reporter Assays for UHRF1 and DNMT1 Inhibition

Cuicui Xia[1,2], Ying Cui[3], Jiongliang Wang[2], Wenjing Bai[2], Danyang Wang[2], Yao Chen[4], Huahui Guo[2], Liangyi Zong[2], Bing Qin[2], Xintong Cai[2], Jie Wang[2], Jing Huang[2], Scott B. Rothbart[5], Zufeng Guo[4,*], Stephen B. Baylin[3,5,*], Xiangqian Kong[1,2,*]

*Correspondence: kong_xiangqian@gibh.ac.cn (X.K.), sbaylin@jhmi.edu (S.B.B.), zfguo@cqmu.edu.cn (Z.G.)

Supplementary information

Figures S1 to S6 and Figure Legends

Tables S3 to S7

Supplementary figures and figure legends

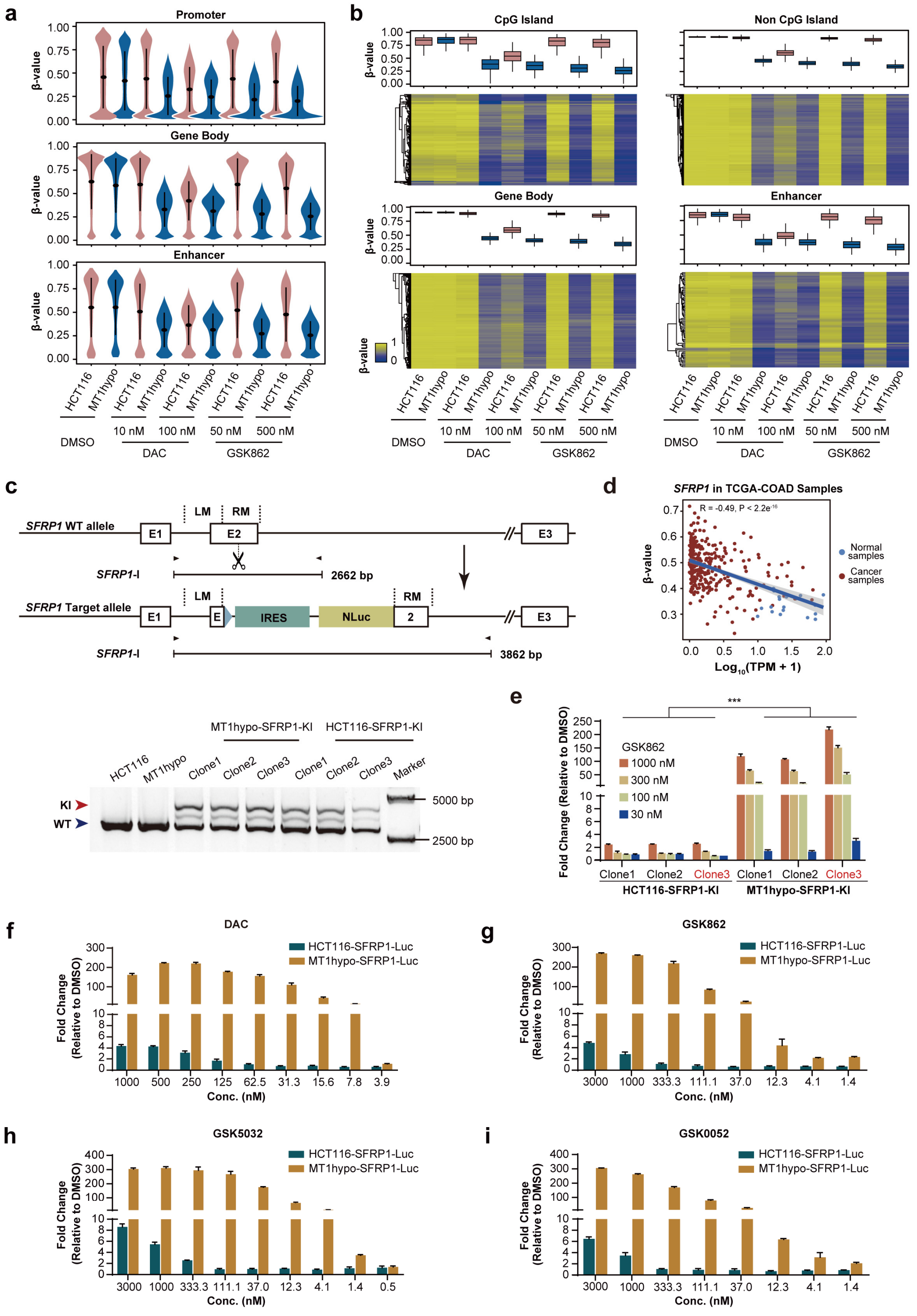

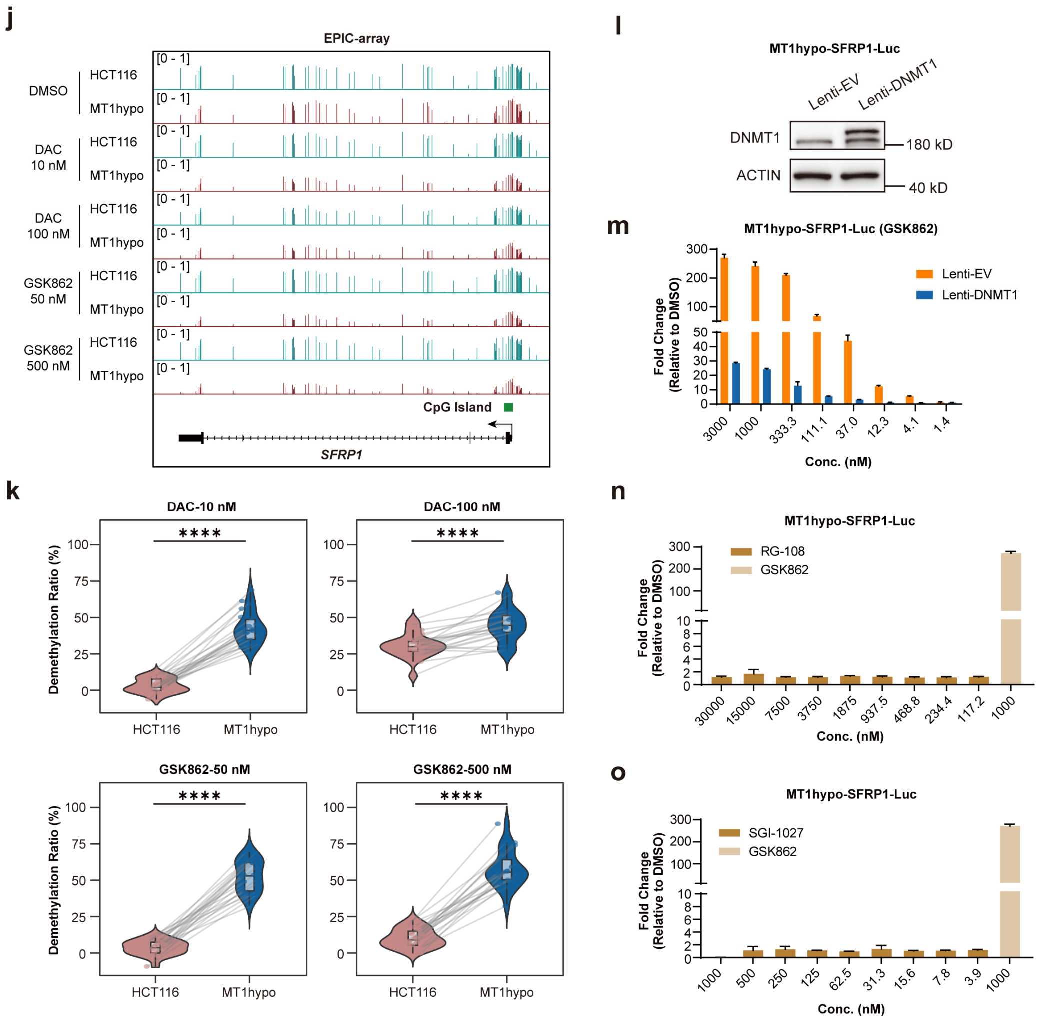

Figure S1. DNMT1 deficiency sensitizes DNA demethylation and reporter activation to DNMT1i. (a) Violin plots displaying DNA methylation (β-value) distributions in the promoter, gene body, and enhancer regions of HCT116 and MT1hypo cells after the indicated treatments for 72 hours. **(b)** DNA demethylation analysis in HCT116 and MT1hypo cells following the indicated treatments for 72 hours. Hypermethylated cancer-specific probes from each region in DMSO-treated cells were used to generate the boxplots and heatmaps. **(c)** PCR validation of successful integration of IRES-NLuc into *SFRP1* locus in HCT116 and MT1hypo-derived cell clones. Successful recombination yielded a 3,862 bp product from the target allele, while the wild-type (WT) allele produced a 2,662 bp fragment. The scissor mark represents the Cas9 cut site. **(d)** Scatterplots showing the correlation between *SFRP1* expression and promoter DNA methylation levels in TCGA-COAD studies. Correlation coefficients (R) and two-sided *p*-values were calculated with Pearson's Correlation. **(e)** NLuc activity in HCT116- and MT1hypo-derived reporter clones measured at 72 hours after GSK862 treatment. The two most responsive clones, highlighted in red, were designated as HCT116-SFRP1-Luc and MT1hypo-SFRP1-Luc, respectively. Statistical significance was determined by two-way ANOVA (***p < 0.001). **(f-i)** Dose-responsive reporter activation by DAC (f), GSK862 (g), GSK5032 (h) and GSK0052 (i) in HCT116-SFRP1-Luc and MT1hypo-SFRP1-Luc cells as depicted in Figure 1e.

(j) DNA methylome (EPIC-Array) profiles at *SFRP1* gene locus in HCT116 and MT1hypo cells following the indicated treatments for 72 hours. CpG island is highlighted in green. **(k)** Violin plots showing differential DNA demethylation across the *SFRP1* promoter following the indicated treatments in HCT116 and MT1hypo cells. All *SFRP1* promoter-associated EPIC array probes were included. Data were normalized to the corresponding DMSO controls, and each point represents a single CpG locus. Statistical significance was determined by a paired two-tailed t-test (****$p < 0.0001$). **(l)** Western blot analysis of DNMT1 protein levels in MT1hypo-SFRP1-Luc cells stably expressing exogenous WT DNMT1. Data are representative of three independent experiments. **(m)** Dose-response analysis of reporter activation in MT1hypo-SFRP1-Luc cells transduced with empty vector (EV) or WT DNMT1 following 72 h treatment with GSK862. **(n-o)** NLuc activity in MT1hypo-SFRP1-Luc cells treated with serially diluted RG-108 (n) or SGI-1027 (o). GSK862 (1000 nM) was used as a positive control. NLuc activity data in (e-i) and (m-o) are presented as mean ± SD (n = 3 technical replicates) and representative of three independent experiments.

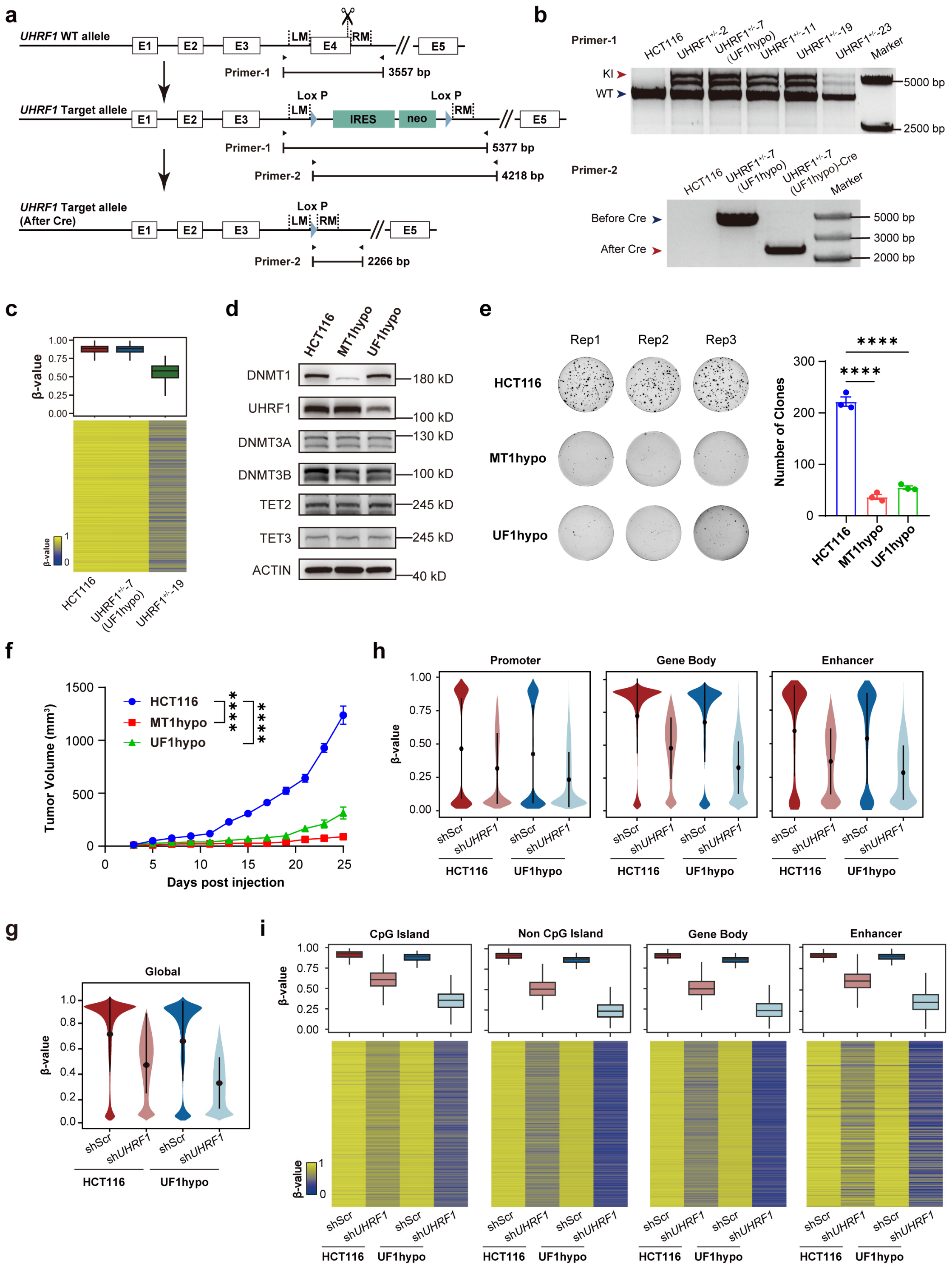

Figure S2. Generation and characterization of UF1hypo cells with increased DNA methylation loss upon further UHRF1 reduction. (a) Schematic of *UHRF1* exon 4 depletion in HCT116 cells. The positions of PCR genotyping primers and expected product sizes are indicated. The scissor mark denotes the Cas9 cut site. **(b)** PCR validation of heterozygous UHRF1 knockout clones. (Top) Confirmation of exon 4 replacement with an IRES-neo cassette from one *UHRF1* allele in selected clones. Successful recombination yielded a 5,377 bp product (red arrow), while the WT allele produced a 3,557 bp fragment (blue arrow). (Bottom)

Verification of IRES-neo cassette excision in UF1hypo cells following Cre expression. Complete excision generated a 2,266 bp product (red arrow), while unexcised alleles remain at 4,218 bp (blue arrow). **(c)** Promoter DNA demethylation analysis in HCT116 cells and two UHRF1$^{+/-}$ clones (UHRF1$^{+/-}$-7 and UHRF1$^{+/-}$-19). Promoter probes with cancer-specific DNA hypermethylation were used to generate the boxplots and heatmaps. **(d)** Western blot analysis of protein levels of major DNA methylation regulators in the indicated cell lines. **(e)** Soft agar assays showing colony formation by HCT116, MT1hypo and UF1hypo cells (left). Colony numbers were quantified using ImageJ and presented as mean ± SEM (n = 3 biological replicates) (right). Statistical significance was determined by an unpaired two-tailed t-test (****$p < 0.0001$). **(f)** Xenograft tumor growth curves following subcutaneous injection of HCT116, MT1hypo, or UF1hypo cells into immunodeficient mice. Data are presented as mean ± SEM (n = 5 mice/group). Tumor volumes at day 25 are compared with HCT116 tumors, and statistical significance was determined by an unpaired two-tailed t-test (****$p < 0.0001$). **(g-h)** Violin plots depicting global (g) and regional (h) DNA methylation levels (β-value) in HCT116 and UF1hypo cells following *UHRF1* knockdown. **(i)** DNA demethylation analysis in HCT116 and UF1hypo cells following *UHRF1* knockdown. Hypermethylated cancer-specific probes from each region were used to generate the boxplots and heatmaps.

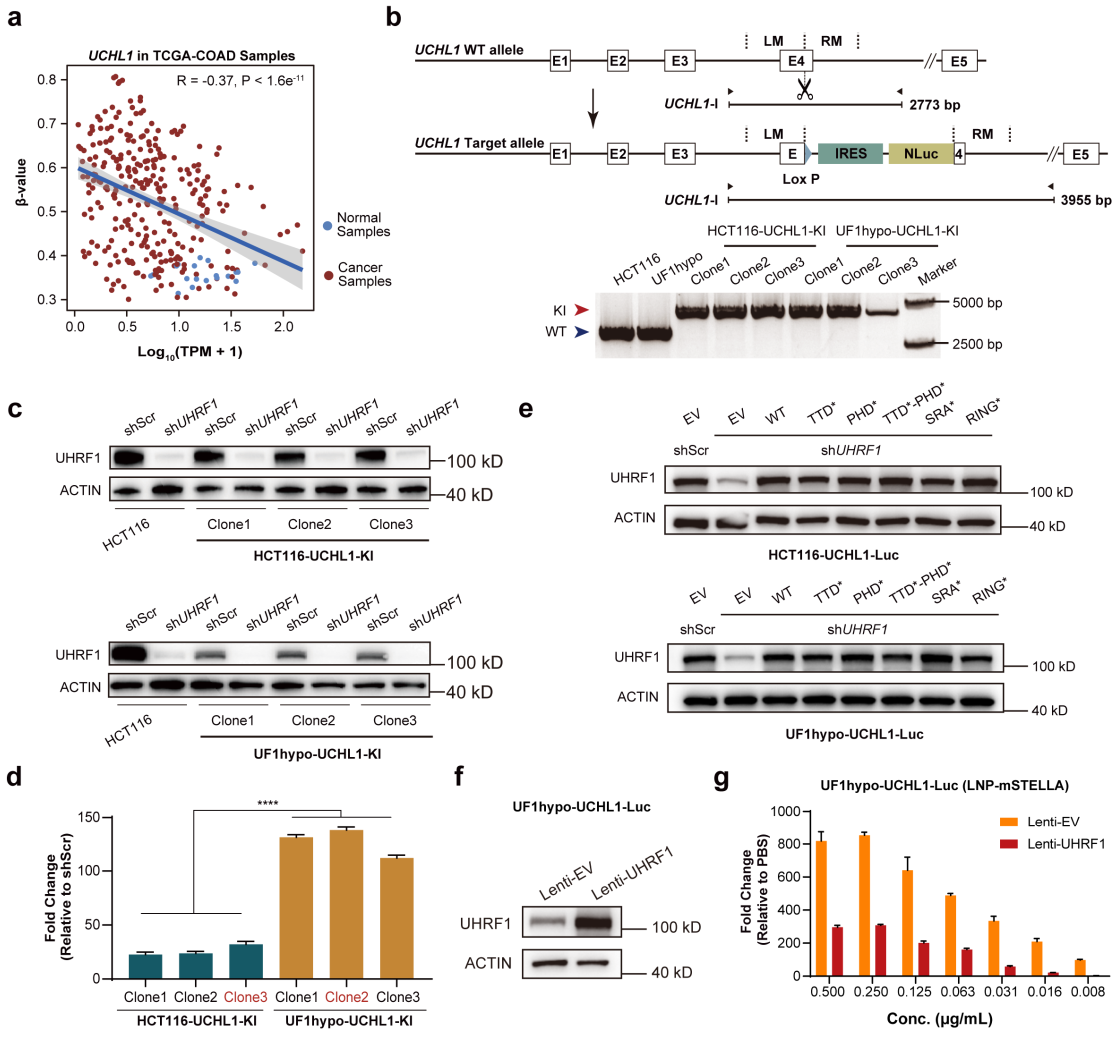

Figure S3. More efficient reporter activation in UHRF1-deficient cell clones compared to cells with full UHRF1 expression. (a) Scatterplots showing the correlation between *UCHL1* expression and promoter methylation levels in TCGA-COAD datasets. Pearson's correlation coefficients (R) and two-sided *p*-values are shown. **(b)** PCR verification of IRES-NLuc knock-in at exon 4 of *UCHL1* in HCT116 and UF1hypo cells. Successful recombination was indicated by a 3,955 bp product (red arrow), while the WT allele produced a 2,773 bp fragment (blue arrow). **(c-d)** Western blot analysis of UHRF1 protein levels (c) and NLuc activation (relative to shScr) (d) in reporter cell clones derived from HCT116 and UF1hypo cells following *UHRF1* knockdown. The two most responsive clones, highlighted in red, were selected for subsequent studies and designated as HCT116-UCHL1-Luc and UF1hypo-UCHL1-Luc, respectively. **(e)** Western blot analysis of UHRF1 protein levels in HCT116-UCHL1-Luc (top) and UF1hypo-UCHL1-Luc (bottom) cells co-expressing indicated UHRF1 domain mutants and shRNA targeting endogenous UHRF1. **(f)** Western blot analysis of UHRF1 protein levels in UF1hypo-UCHL1-Luc cells stably expressing exogenous WT UHRF1. **(g)** Dose-responsive reporter activation in UF1hypo-UCHL1-Luc cells transduced with EV or WT UHRF1 following 72 h treatment with LNP-mSTELLA. Western blots in (c), (e) and (f) are representative of three independent experiments. NLuc activity data in (d) and (g) are presented as mean ± SD (n = 3

technical replicates) and representative of three independent experiments. Statistical significance was determined by two-way ANOVA (****$p < 0.0001$).

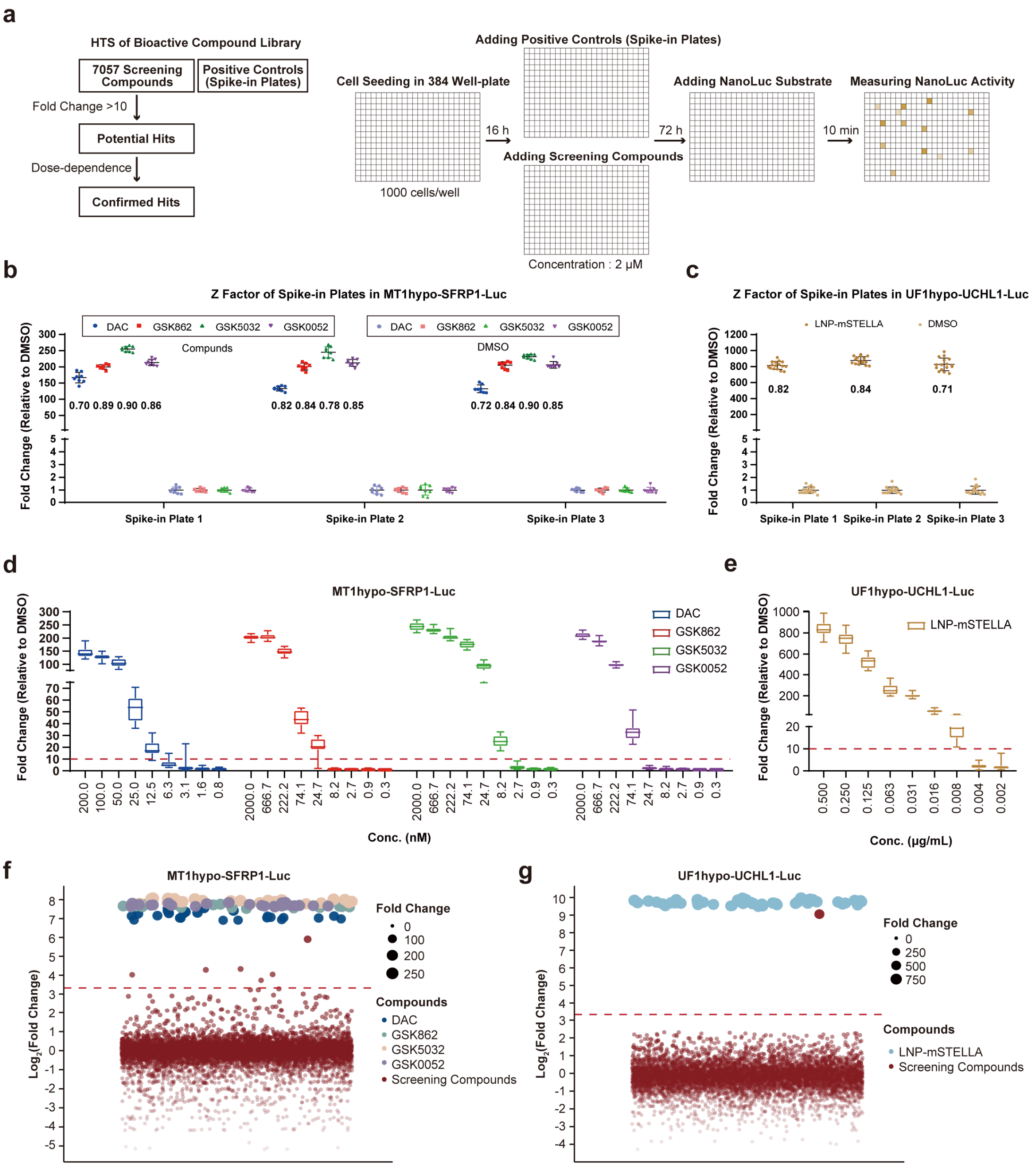
a
HTS of Bioactive Compound Library
7057 Screening Compounds
Positive Controls (Spike-in Plates)
Fold Change >10
Potential Hits
Dose-dependence
Confirmed Hits
Adding Positive Controls (Spike-in Plates)
Cell Seeding in 384 Well-plate
1000 cells/well
16 h
Adding Screening Compounds
Concentration : 2 μM
72 h
Adding NanoLuc Substrate
10 min
Measuring NanoLuc Activity
b
Z Factor of Spike-in Plates in MT1hypo-SFRP1-Luc
DAC GSK862 GSK5032 GSK0052
Compunds
DMSO
0.70 0.89 0.90 0.86
0.82 0.84 0.78 0.85
0.72 0.84 0.90 0.85
Fold Change (Relative to DMSO)
Spike-in Plate 1
Spike-in Plate 2
Spike-in Plate 3
c
Z Factor of Spike-in Plates in UF1hypo-UCHL1-Luc
LNP-mSTELLA DMSO
0.82
0.84
0.71
d
MT1hypo-SFRP1-Luc
DAC
GSK862
GSK5032
GSK0052
Conc. (nM)
e
UF1hypo-UCHL1-Luc
LNP-mSTELLA
Conc. (μg/mL)
f
MT1hypo-SFRP1-Luc
Log2(Fold Change)
Fold Change
0
100
200
250
Compounds
DAC
GSK862
GSK5032
GSK0052
Screening Compounds
g
UF1hypo-UCHL1-Luc
Fold Change
0
250
500
750
Compounds
LNP-mSTELLA
Screening Compounds

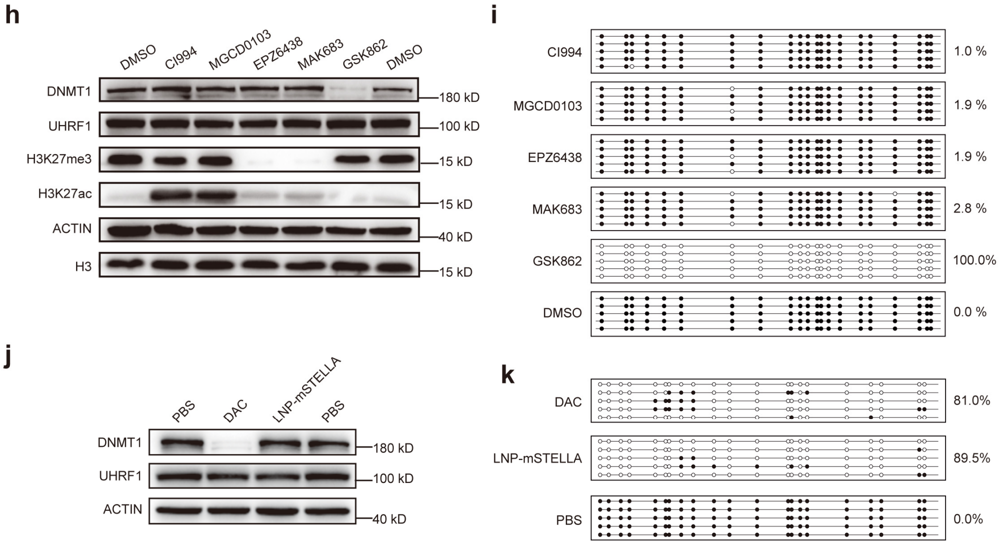

Figure S4. Hit identification and validation for the HTS of a Bioactive Compound Library. **(a)** (Left) Schematic illustrating the HTS using optimized reporter cells. A library of 7,057 compounds was screened in parallel with spike-in reference controls. Primary hits were defined as compounds inducing > 10-fold change NLuc activation compared to untreated controls, followed by dose-response validation. (Right) Experimental setup of the HTS. Reporter cells (1,000 per well) were seeded in 384-well plates, incubated for 16 hours, then treated with compounds at 2 μM or serially diluted reference compounds using automated robotics. NLuc activity was measured 72 hours post-treatment after a 10-minute incubation with detection reagent, with fold change calculated relative to untreated controls. **(b)** Z factor calculation using 200 nM DAC, 2000 nM GSK862, GSK5032 or GSK0052 (n = 8 technical replicates) in each spike-in plate for MT1hypo-SFRP1-Luc-based HTS. **(c)** Z factor calculation using 0.5 μg/mL LNP-mSTELLA (n = 16 technical replicates) in each spike-in plate for UF1hypo-UCHL1-Luc-based HTS. **(d)** Dose-response NLuc activation of the reference DNMT1i in three spike-in plates for MT1hypo-SFRP1-Luc. Boxplots show NLuc activity induced by DAC (starting at 200 nM with 2-fold serial dilutions), or GSK862, GSK5032, GSK0052 (starting at 2000 nM with 3-fold serial dilutions). The box represents the interquartile range, the line inside indicates the median, and the whiskers extend to the minimum and maximum values (n = 24 from three spike-in plates). **(e)** Dose-response NLuc activation of the reference UHRF1i in three spike-in plates for UF1hypo-UCHL1-Luc. Boxplots show NLuc activity induced by LNP-mSTELLA (starting at 0.5 μg/mL with 2-fold serial dilutions), represented as in (D) (n = 48 from three spike-in plates). **(f-g)** Scatterplots of NLuc activity in MT1hypo-SFRP1-Luc (f) and UF1hypo-UCHL1-Luc (g) cells treated with reference and screening compounds. Each dot represents a compound, with size proportional to fold change. The red dashed lines indicate the threshold for selection (Fold Change > 10). Reference compounds in (f) include DAC (200 nM) and GSK inhibitors (2000 nM; n = 24); in (g), LNP-mSTELLA (0.5 μg/mL; n = 48). **(h)** Western blot analysis of DNMT1, UHRF1, H3K27me3, and H3K27ac protein levels in MT1hypo-SFRP1-Luc cells treated with CI994, MGCD0103, EPZ6438, MAK683, or GSK862 (2000 nM) for 12

hours. **(i)** Bisulfite sequencing-based DNA methylation analysis of the *SFRP1* promoter region in MT1hypo-SFRP1-Luc cells treated with CI994 (2000 nM), MGCD0103 (300 nM), EPZ6438, MAK683, or GSK862 (2000 nM) for 72 hours. **(j)** Western blot analysis of DNMT1 and UHRF1 levels in UF1hypo-UCHL1-Luc cells treated with DAC (2000 nM) or LNP-mSTELLA (0.2 μg/mL) for 12 hours. **(k)** Bisulfite sequencing-based DNA methylation analysis of the *UCHL1* promoter region in UF1hypo-UCHL1-Luc cells treated with DAC (500 nM) or LNP-mSTELLA (0.2 μg/mL) for 72 hours. Western blots in (h) and (j) are representative of three independent experiments.

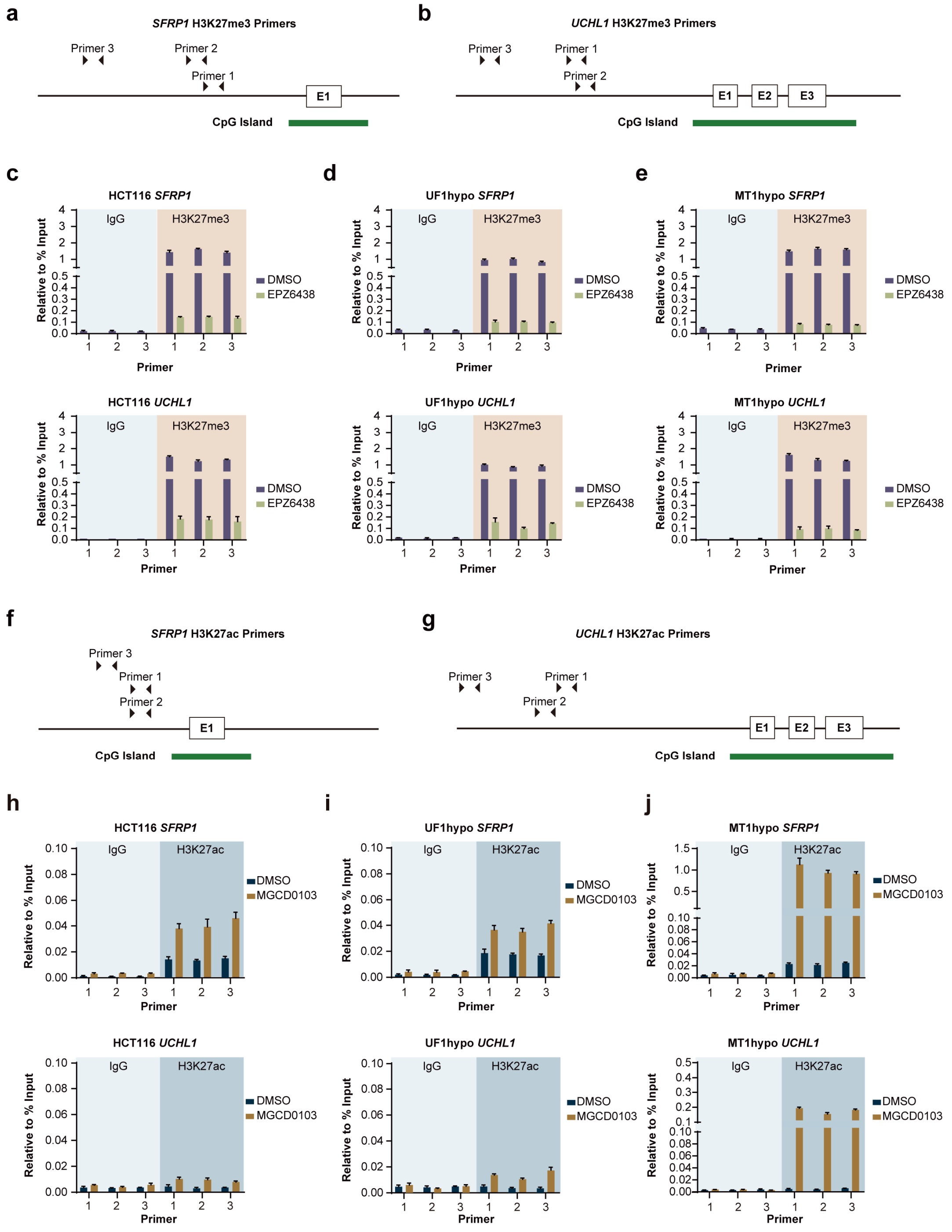
a
SFRP1 H3K27me3 Primers
Primer 3
Primer 2
Primer 1
E1
CpG Island
b
UCHL1 H3K27me3 Primers
Primer 3
Primer 1
Primer 2
E1
E2
E3
CpG Island
c
HCT116 SFRP1
HCT116 UCHL1
d
UF1hypo SFRP1
UF1hypo UCHL1
e
MT1hypo SFRP1
MT1hypo UCHL1
IgG
H3K27me3
DMSO
EPZ6438
Relative to % Input
Primer
f
SFRP1 H3K27ac Primers
Primer 3
Primer 1
Primer 2
E1
CpG Island
g
UCHL1 H3K27ac Primers
Primer 3
Primer 1
Primer 2
E1
E2
E3
CpG Island
h
HCT116 SFRP1
HCT116 UCHL1
i
UF1hypo SFRP1
UF1hypo UCHL1
j
MT1hypo SFRP1
MT1hypo UCHL1
IgG
H3K27ac
DMSO
MGCD0103
Relative to % Input
Primer

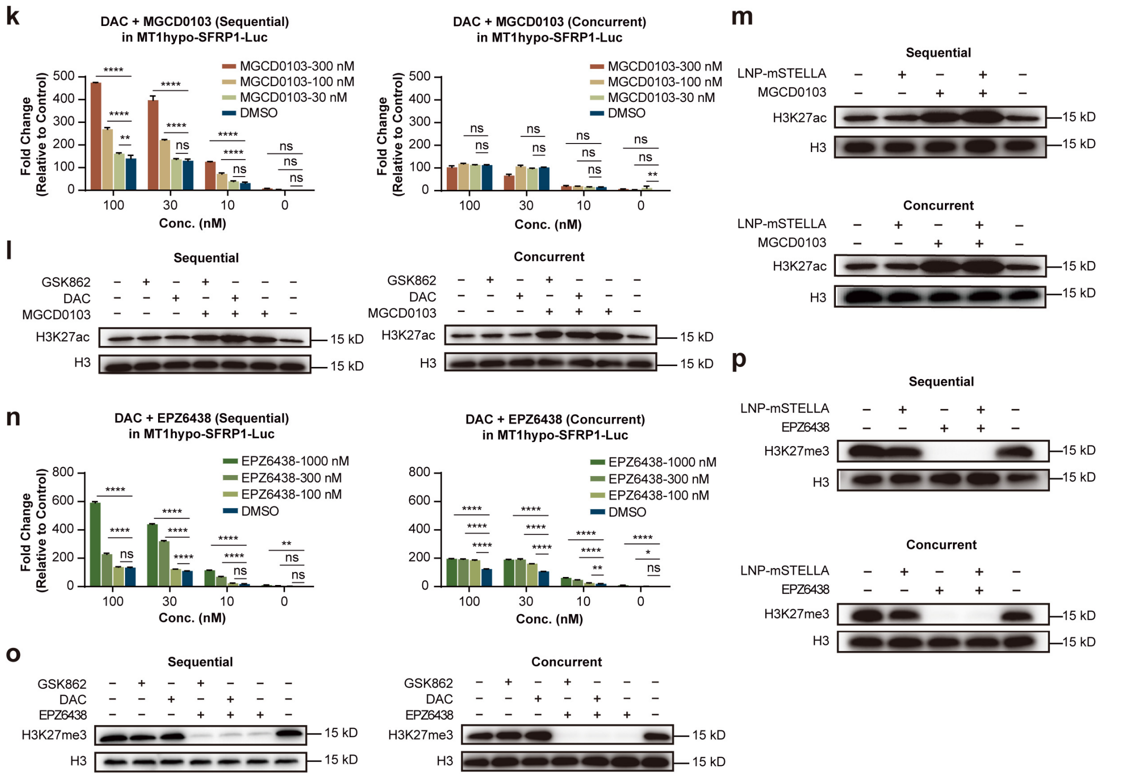

Figure S5. Combining EZH2i or HDACi with DNA methylation inhibitors enhanced reporter re-expression. (a-b) Schematic of primer design for H3K27me3 ChIP-qPCR. Three primer pairs were designed to amplify regions upstream of CpG islands (green boxes) at the *SFRP1* (a) and *UCHL1* (b) loci. **(c-e)** H3K27me3 enrichment at the *SFRP1* and *UCHL1* loci in HCT116 (c), UF1hypo (d), and MT1hypo (e) cells after 72-hour treatment with EPZ6438 or DMSO. **(f-g)** Schematic of primer design for H3K27ac ChIP-qPCR targeting upstream regions of CpG islands at the *SFRP1* (f) and *UCHL1* (g) loci. **(h-j)** H3K27ac enrichment at the same loci in HCT116 (h), UF1hypo (i), and MT1hypo (j) cells after 72-hour treatment with MGCD0103 or DMSO. **(k)** NLuc activity in MT1hypo-SFRP1-Luc cells following sequential or concurrent treatment with DAC and MGCD0103. Control indicates cells treated with equivalent volumes of DMSO and PBS (vehicle for DAC). **(l-m)** Western blot analysis of H3K27ac levels after sequential or concurrent treatment with DNMT1i (l) or UHRF1i (m) combined with MGCD0103 (corresponding to Figures 5d-5e and S5k). **(n)** NLuc activity in MT1hypo-SFRP1-Luc cells following sequential or concurrent treatment with DAC and EPZ6438. Controls are as described in (k). **(o-p)** Western blot analysis of H3K27me3 levels after sequential or concurrent treatment with DNMT1i (o) or UHRF1i (p) combined with EPZ6438 (corresponding to Figures 5f-5g and S5n). ChIP-qPCR data in (Figures S5c-e and S5h-j) are presented as mean ± SD (n = 3 technical replicates) and representative of two independent experiments. Data in (k) and (n) are presented as mean ± SD (n = 3 technical replicates) and representative of three independent experiments. Western blots are representative of three independent experiments. Statistical significance was assessed by two-way ANOVA (**p* < 0.05; ***p* < 0.01; ****p* < 0.001; *****p* < 0.0001; and "ns" for not

significant).

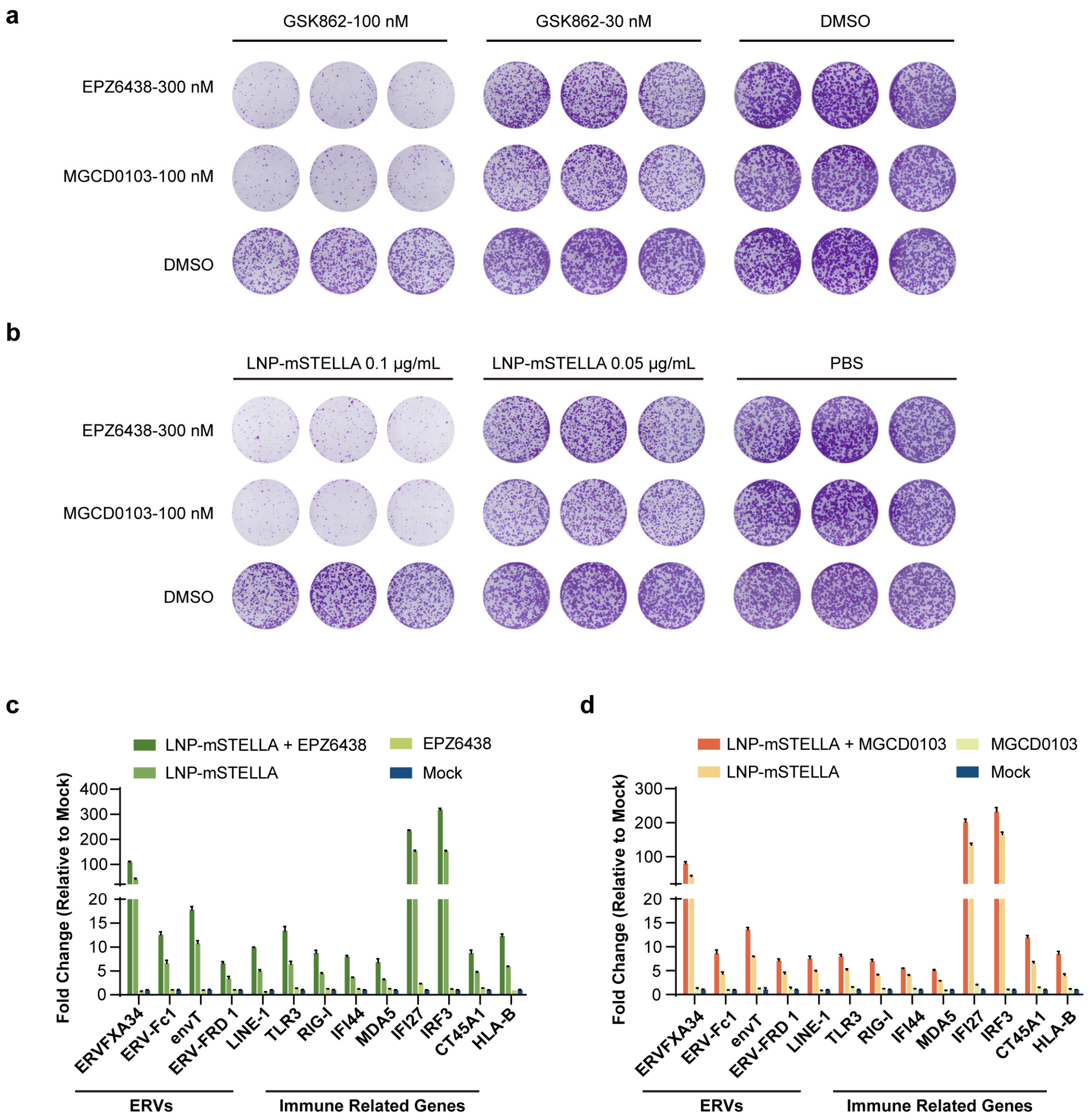

Figure S6. Sequential epigenetic treatments enhances CRC cell growth inhibition and induction of viral-mimicry associated immune genes. (a-b) Colony formation of HCT116 cells following sequential treatment with GSK862 (a) or LNP-mSTELLA (b) and histone modification inhibitors. Cells were treated with GSK862 (a) or LNP-mSTELLA (b) for 3 days, followed by MGCD0103 or EPZ6438 for 2 days. Pre-treated cells were then seeded into 6-well plates and cultured for an additional 7 days before crystal violet staining. Data are representative of three independent experiments. **(c-d)** qPCR analysis of endogenous retroviruses (ERVs) and immune-related genes in HCT116 cells with the indicated treatments. Cells were treated sequentially with 0.2 μg/mL LNP-mSTELLA for 3 days, followed by 100 nM EPZ6438 or 30 nM MGCD0103 for an additional 2 days. DMSO and PBS served as vehicle controls (Mock). Data are presented as mean ± SD (n = 3 technical replicates) and representative of three independent experiments.

Supplementary Table S3-S7

Table S3 Sequences of shRNA and sgRNA

Gene	Sequence
**shRNA**	
UHRF1	CCGGGCCTTTGATTCGTTCCTTCTTCTCGAGAAGAAGGAACGAATCAAAGGCTTTTTG (**TRCN0000273256**)
**sgRNA**	
SFRP1	sense: 5'-caccgGTGACAACGAGTTGAAATCTG-3'<br>antisense: 5'-aaacCAGATTTCAACTCGTTGTCACc-3'
UHRF1	sense: 5'-caccgCGTGGTCCAGATGAACTCCA-3'<br>antisense: 5'-aaacTGGAGTTCATCTGGACCACGc-3'
UCHL1	sense: 5'-caccgAATCGGACTTATTCACGCAG-3'<br>antisense: 5'-aaacCTGCGTGAATAAGTCCGATTc-3'

Table S4 Primers used for genotyping of knock-in clones

Gene	Forward primer	Reverse primer
SFRP1-I	TGGAAGGTGTTTGCTCCTGG	TACGCGCTCTCCAGTTTAGC
UHRF1-primer-1	GTCTCCAGTCTTTGCACAAGT	TCCCAGCCCAGGACCTCTGC
UHRF1-primer-2	AGTGGATCCATAACTTCGTATAGCA	ACAAAAATTAGCTGGTGGTGG
UCHL1-I	CGAGCGCCTATTCTGCTACAC	TTACCCGACATTGGCCTTCC

Table S5 Primers for Real-time qPCR analysis

Gene	Forward primer	Reverse primer
UCHL1	AGAATGAGGCCATACAGGCAG	GGTGGCCATCCACGTTGTTA
SFRP1	TGGCCCGAGATGCTTAAGTG	GACACACCGTTGTGCCTTG
FBLN2	TGGCGTGTCCTGTGAAGACAT	GAGTGCCTTGTAGCAGTGGAA
LXN	ACAAGCCAGCATGGAGGATA	TCAGCTGTGCAGTTCACCTT
ICAM4	AGGATCACCGCCTACAAACC	GTGGCAGCGCAAAGTGTATT
β-Actin	CAACCGCGAGAAGATGACC	TAGCACAGCCTGGATAGCAA
ERVFXA34	CAGGAAACTAACTTTCAGCCAA	TAAAGAGGGCATGGAGTAATTGA
ERV-Fc1	TACACCCTTACTCCCGTCTT	GCCTAACATTCCGACCTCATAC
envT	AGGATTTGATGTTGGGACTATGTT	GGTGTTTCTGGAATATAGGGTCAC
ERV-FRD 1	AGCCAGCTCTCAAAGGAAATAG	GAAGGACTACGGCTGCTAAAG
TLR3	TGGTTGGGCCACCTAGAAGTA	TCTCCATTCCTGGCCTGTG
RIG-I	CCAGCATTACTAGTCAGAAGGAA	CACAGTGCAATCTTGTCATCC
IFI44	TGGGAGCTGGACCCTGTAAA	CCTCCCTTAGATTCCCTATTTGCT
MDA5	GAGCAACTTCTTTCAACCACAG	CACTTCCTTCTGCCAAACTTG
IFI27	ATCAGCAGTGACCAGTGTGG	TGGCCACAACTCCTCCAATC
CT45A1	GCACCTGTGGGAGGAAACG	CCTGACTGCAGTAGGTCCTTG
HLA-B	CCTGAGATGGGAGCCGTCTT	CTCCGATGACCACAACTGCT
LINE-1	GCCAAGATGGCCGAATAGG	TGGCACTCCCTAGTGAGATGAA
IRF3	TCTGCCCTCAACCGCAAAGAAG	TACTGCCTCCACCATTGGTGTC

Table S6 Primers for BSP analysis

Gene	Forward primer	Reverse primer
SFRP1	AGGTAGTTTTATTTTGGGGTTTG	ACACCCAAATCTTCCTCTACTC
UCHL1	GAGTTTGGGAGGAGTTTTAGTTT	CTTTCCTAAACCCCTACATTTAAC

Table S7 Primer sequences used for ChIP-qPCR analysis of *SFRP1* and *UCHL1*

For H3K27me3 enrichment		
	**Forward primer**	**Reverse primer**
SFRP1		
Primer 1	TGGCCAATGGAAGATGCCTT	ATTAACCCTTGGCCATCCCG
Primer 2	CGAATGGCAGGAGGACTTGT	GTGACCAGACATAGGCGAGG
Primer 3	AGGGGCCAAAGTGGTATTCG	GTTAGCAGGTCAGGCCAGTT
UCHL1		
Primer 1	CAGTGGTTTCCTTGGTTGCC	CAAAGAAGGCAGGCGATTGG
Primer 2	TTCCGCGGGAGTCACATTAC	GACCAGTACCGAGGTTCGTT
Primer 3	TGGGATTCTGATCTGTAAAATTGC	TGGGGATCCTTTGCATGTGT
**For H3K27ac enrichment**		
	**Forward primer**	**Reverse primer**
SFRP1		
Primer 1	TCAGCGTGGCTGGTGTAAAT	CTGATACCGTCCTCTTGGCG
Primer 2	ATCAGCGTGGCTGGTGTAAA	CTGATACCGTCCTCTTGGCG
Primer 3	GGGAGTGAGACCCGGACTAT	CGCGCCCTATAGTGTCTCTG
UCHL1		
Primer 1	TTGGAGCCCAGTTTAGCAGG	AGAGGAGCCGTGAAGAGACT
Primer 2	TCACTTGTCTTGACCTTCACCT	TGACCTTCACACACAAAAATGAGA
Primer 3	ATCCCCTCCACTAGGTTAGGT	CCCTCGTGCTGTTTTTATCAGT
